# Supplementary material for: Allosteric regulation and crystallographic fragment screening of SARS-CoV-2 NSP15 endoribonuclease
Source: Nucleic Acids Res. 2023 Apr 28;51(10):5255–70. doi: 10.1093/nar/gkad314 (PMC10250223; doi:10.1093/nar/gkad314)
Supplement: gkad314_Supplemental_Files [file gkad314_supplemental_files.zip › Supplementary datasets.zip!/159/Refine_4/01-BUSTER/Cycle-4/LIST.html]

Refining

**BUSTER 2.X**: A suite of programs for macromolecular structure refinement by maximum-likelihood and Bayesian methods  
Copyright © 1992-2020 Global Phasing Ltd.  
All rights reserved.  
Authors: G. Bricogne, E. Blanc, M. Brandl, C. Flensburg, P. Keller, W. Paciorek, P. Roversi, O. Smart, C. Vonrhein, T. Womack  

Please reference use of the software as:

- BUSTER-TNT 2.X, Global Phasing Ltd, Sheraton House, Cambridge CB3 0AX, UK

Version: 2.10.3 for linux64-ifort made on May 20 2020 at 01:14:34  
Licenced to: Diamond Light Source, Scientific Software  

```
---


### Parsing of the BUSTER cardfile

TITLE : autoBUSTER refinement

   Cell information

   Symmetry information

 Using CCP4 X-ray form factors.

   Missing structure chemical composition

 Using ATOM block for chemical composition.


   Shell information

### Parsing of the TNT cardfile


---


### Reading observations file

 Number of reflexions in input:  91725

      Number of accepted reflexions:  85472

      Number of rejected reflexions:      0

   Reflexions in test set:            4245

   Reflexions in working set:        81227


---


### 1. Generating initial model

 2.10 * Shannon sampling rate. NX NY NZ:   360  360  240


  Effective Shannon rates are:    2.43    2.43    2.22


   Allocated BUFFER of size:    119.312 Mb.

   Initial Geometry Information


 No missing atoms declared


   Summary of model volumes

 No missing atoms declared: no prior computed


 Computing bulk solvent model scattering


 NCSMASK PDB radius for solvent mask:     2.15 Angstrom

 running PDBSET (setting cell and spacegroup P1) ... done
 running NCSMASK [1] (creating mask, SMOOTH 0.923692) ... done
 running MAPDUMP (to check if reduction to unit cell is needed) ... done
 running MAPMASK (converting mask to map) ... done

   Generation of babslv by theta-filter

   Electron density in frg mask:  0.420 Å-1

 FOBS  put on rough absolute scale by Wilson plot :
           K        =  0.46946E+02
           B        =     -6.800

 

### Cycle 0001: MaxLik scaling and refinement


   MaxLik Scaling Cycle 0001

   Wilson plot

   Statistics in resolution bins just after scaling
```

|  |  |
| --- | --- |
|  | - Rxpct factors plot- Rxpct factors table- <Log-Lik Gain>- Ln Sigma A- Correlation Coefficients |

```
   <Log-Likelihood> of null-hypothesis

Structure Factor Amplitudes


   Average Figure of Merit

 Switching off refinement of overall temperature factor


 Added B =     0.4417 Å2 to all atoms in fragment


 Added B =     0.4417 Å2 to B_SOLV

```
gelly refinement module
 Authors (2004-2009) O. S. Smart, C. Flensburg, W. Paciorek, C. Vonrhein, 
                     M. Brandl, T. O. Womack and G. Bricogne
         (2010-2011) O. S. Smart, C. Flensburg, W. Paciorek, C. Vonrhein, 
                     T. O. Womack and G. Bricogne
         (2012-2014) O. S. Smart, C. Flensburg, W. Paciorek, C. Vonrhein, 
                     and G. Bricogne
         (2015-2020) G. Bricogne, C. Flensburg, W. Paciorek, C. Vonrhein
 Copyright © 2004-2020 Global Phasing Ltd. 
 All rights reserved.
 gelly module compilation date May 20 2020 01:11:20
 
 gelly uses routines from TNT geometry. References: 
     Tronrud, D. E., Ten Eyck, L. F., Matthews, B. W. (1987)
     "An Efficient General-Purpose Least-Squares Refinement Program 
     for Macromolecular Structures". Acta Crystallogr A, 43:489-501.
     Tronrud, D. E. (1997). "The TNT Refinement Package", 
     in Macromolecular Crystallography, Part B, Eds Charlie Carter 
     and Robert Sweet, Methods in Enzymology, 277:306-319.
 wildcard matching function by Julian Robichaux http://www.nsftools.com

 No -random_seed flag found. Random number stream initialised using:
2147483562
2147483398

 Control flags set to:  -autoncs -hydrogens_input_occ2zero \
                     -hydrogens_output_occ2heavy -screen \
                     100 -screen_sigma \
                     3.0 -keeppdb \
                     1000000 -taomonitor_verbose \
                     2 
 

 ############### Starting setup via TNT routines

 Setting up tnt routines including license check:

                   TNT Stereochemistry Program
 Version: 5.22.3.0
 Licence for Diamond Light Source, Scientific Software is valid
 
 Using TNT geometry routines to load the tnt control file: gelly.tnt
 Using TNT routines to load initial coordinates from file fragment.cor
 Expecting 6118 atoms

 Found     2189 "NOTE BUSTER_RESIDUE_ATOM_DATA" cards
 Have copied information for    6118 atoms from TNT to gelly's store 
 
 The TNT WEIGHT cards used by this run:
 -----WEIGHT       BOND     2.0
 -----WEIGHT       ANGLE    2.0
 -----WEIGHT       IMPROPER 0.0
 -----WEIGHT       TORSION  2.0
 -----WEIGHT       PSEUDO   0.0
 -----WEIGHT       TRIGONAL 2.0
 -----WEIGHT       PLANE    5.0
 -----WEIGHT       CONTACT  5.0
 -----WEIGHT       BCORREL  10.0
 -----WEIGHT       CHIRAL   5.0
 -----WEIGHT       NCS      50.0
 -----WEIGHT       IDEAL    4.0
 -----WEIGHT       RFACTOR  5.44
 
 Dictionary versions picked up from NOTE BUSTER_RESTRAINT_DICT cards as: 
 ------ protgeo_eh99.dat (V1.6.4.17) 20191001  STANDARD AMINO ACID DICTIONARY. BONDS AND ANGLES FROM ENGH AND HUBER EH99. OTHER VALUES BASED ON PREVIOUS TNT OR TAKEN FROM CCP4. INCLUDES HYDROGEN ATOMS.
 ------ exoticaa.dat (V1.3.4.15) 20200327  COLLECTION OF NON-STANDARD AMINO ACIDS, MAINLY EH91 WITHOUT IDEAL DISTANCE INFO
 ------ nuclgeo.dat (V1.13.4.8) 20190909
 ------ bcorrel.dat (V1.15.10.2) 20190616
 ------ contact.dat (V1.15.12.10) 20191121
 ------ idealdist_contact.dat (V1.3.4.12) 20191001  IDEAL-DISTANCE CONTACT TERM DATA AS USED IN PROLSQ. VALUES USED HERE ARE BASED ON DATA HARVESTING OF PDB AS OF 20190109.
 ------ restraints for LIG (Unknown) from cif dictionary Z1437171658.cif
 ------ restraints for CIT (CITRIC ACID) from cif dictionary CIT.cif; generated by GRADE_PDB_LIGAND 1.2.12 (October 4 2016) using MOGUL 1.7.2(RC1), CSD as538be, with quantum mechanics RM1 ((DatabaseID PDB-CIT))
 ------ assume.dat (V1.9.4.2) 20180705
 
 Have picked up spacegroup name for pdb output as 'P 63      '
 Setting up Chain/Residue arrays
 
 Have command line specification -autoncs*
     Have found       1 NCS relations
     Pairwise matching residue number and type between chains: 
                    A        B        S    
          A         .       99%       -   
          B                  .        -   
          S                           .   
       Table key--                                                       
          all   : two chains have 100% match and same number of residues 
         multi  : ditto and they contribute to a multi chain ncs cluster 
          ***%  : two chains have >80% matching residue number/type      
           -    : chains appear to be unrelated                          
      chain A     have identified ncs to     1 other chains. So will weight each autoncs by (       6.666/#)=      6.6660
      chain B     have identified ncs to     1 other chains. So will weight each autoncs by (       6.666/#)=      6.6660
      chain S     have identified ncs to     0 other chains. 
     For .Gelly cards produced see below - after pruning
 
 Setting up BUSTER_SET groups
 Have found    89 BUSTER_SET cards
 Now silently applying automatic/dictionary set definitions. For a verbose listing use '-verbose_set' flag.
 Applying user defined set definitions:
 =====NOTE BUSTER_SET ncsautoXcld = Water --> set 'ncsautoXcld' now has   581 atoms 
 =====NOTE BUSTER_SET ncsautoABset = Chain_A \ ncsautoXcld --> set 'ncsautoABset' now has  2770 atoms 
 Have defined    48 sets: 
      set:                 'All' has   6118 atoms in   1281 residues - automatically defined
      set:               'Empty' has      0 atoms in      0 residues - automatically defined
      set:            'ExoticAA' has      0 atoms in      0 residues - defined by dictionary file
      set:           'ExoticNUC' has      0 atoms in      0 residues - defined by dictionary file
      set:              'NotHET' has   5514 atoms in    698 residues - defined by dictionary file
      set:             'Chain_A' has   2770 atoms in    350 residues - automatically defined
      set:             'Chain_B' has   2767 atoms in    350 residues - automatically defined
      set:             'Chain_S' has    581 atoms in    581 residues - automatically defined
      set:               'Water' has    581 atoms in    581 residues - defined by dictionary file
      set:          'StdProtein' has   5514 atoms in    698 residues - defined by dictionary file
      set:             'Protein' has   5514 atoms in    698 residues - defined by dictionary file
      set:                'Back' has   2794 atoms in    698 residues - defined by dictionary file
      set:                'Side' has   2720 atoms in    650 residues - defined by dictionary file
      set:        'RnaCanonical' has      0 atoms in      0 residues - defined by dictionary file
      set:        'DnaCanonical' has      0 atoms in      0 residues - defined by dictionary file
      set:     'RnaDnaCanonical' has      0 atoms in      0 residues - defined by dictionary file
      set:     'DnaRnaCanonical' has      0 atoms in      0 residues - defined by dictionary file
      set:         'RnaExtended' has      0 atoms in      0 residues - defined by dictionary file
      set:         'DnaExtended' has      0 atoms in      0 residues - defined by dictionary file
      set:      'RnaDnaExtended' has      0 atoms in      0 residues - defined by dictionary file
      set:      'DnaRnaExtended' has      0 atoms in      0 residues - defined by dictionary file
      set:                 'Dna' has      0 atoms in      0 residues - defined by dictionary file
      set:                 'Rna' has      0 atoms in      0 residues - defined by dictionary file
      set:              'RnaDna' has      0 atoms in      0 residues - defined by dictionary file
      set:              'DnaRna' has      0 atoms in      0 residues - defined by dictionary file
      set:             'Nucleic' has      0 atoms in      0 residues - defined by dictionary file
      set:             'Polymer' has   5514 atoms in    698 residues - defined by dictionary file
      set: 'BufferWeichenberger' has     13 atoms in      1 residues - defined by dictionary file
      set:    'BufferAdditional' has      0 atoms in      0 residues - defined by dictionary file
      set:              'Buffer' has     13 atoms in      1 residues - defined by dictionary file
      set:         'MetalAlkali' has      0 atoms in      0 residues - defined by dictionary file
      set:  'MetalAlkalineEarth' has      0 atoms in      0 residues - defined by dictionary file
      set:     'MetalTransition' has      0 atoms in      0 residues - defined by dictionary file
      set: 'MetalPostTransition' has      0 atoms in      0 residues - defined by dictionary file
      set:    'MetalLanthanides' has      0 atoms in      0 residues - defined by dictionary file
      set:               'Metal' has      0 atoms in      0 residues - defined by dictionary file
      set:         'SugarAlphaD' has      0 atoms in      0 residues - defined by dictionary file
      set:         'SugarAlphaL' has      0 atoms in      0 residues - defined by dictionary file
      set:               'Sugar' has      0 atoms in      0 residues - defined by dictionary file
      set:               'Other' has     23 atoms in      2 residues - defined by dictionary file
      set:            'Hydrogen' has      0 atoms in      0 residues - defined by dictionary file
      set:         'nonHydrogen' has   6118 atoms in   1281 residues - defined by dictionary file
      set:             'OccZero' has      0 atoms in      0 residues - defined by dictionary file
      set:          'OccNonZero' has   6118 atoms in   1281 residues - defined by dictionary file
      set:             'OccFull' has   6076 atoms in   1278 residues - defined by dictionary file
      set:          'OccNonFull' has     42 atoms in      3 residues - defined by dictionary file
      set:         'ncsautoXcld' has    581 atoms in    581 residues - user specified
      set:        'ncsautoABset' has   2770 atoms in    350 residues - user specified
 No command line arguments '-occ_allhet' or '-occ_restyp' so will not setup automated occcupancy refinement
 
 Interpreting CONSTANT and FREE cards: 
 =====CONSTANT     OCC
 Have found and interpreted      1 CONSTANT cards in the TNT input. 
 Before any COMBINE - Number of atoms with fixed XYZ =     0
 Before any COMBINE - Number of atoms with fixed B   =     0
 Before any COMBINE - Number of atoms with fixed OCC =  6118
 Before any COMBINE - Number of atoms with fixed BIJ =   581
                            cf total number of atoms =  6118
 
 There are no COMBINE cards to be found in the TNT card input 
 
 There are no NOTE BUSTER_COMBINE_RES cards to be found in the TNT card input 

 ############### Working out full connectivity list


 ############### Working out equivalent atoms


 Residue with most atoms given below:
        A|58:N(TRP)         
        A|58:CA(TRP)        
        A|58:CB(TRP)        
        A|58:CG(TRP)        
        A|58:CD1(TRP)       
        A|58:NE1(TRP)       
        A|58:CE2(TRP)       
        A|58:CD2(TRP)       
        A|58:CE3(TRP)       
        A|58:CZ3(TRP)       
        A|58:CH2(TRP)       
        A|58:CZ2(TRP)       
        A|58:C(TRP)         
        A|58:O(TRP)         


 Have no '-equiv_check_plus' argument - so will take atom type into
 account when checking for equivalence of otherwise identical atoms.
 

 ############### Looking at dihedrals


 ############### Looking at chirals

 NOTE BUSTER_CHIRAL_ANGLECUT card found - read value=    -31.00 degs from it
 NOTE BUSTER_CHIRAL_SIGMA card found - read value=      1.70 degs from it
 RANGE allowed for B variables in refinement:   from      3.00000 to    300.00000

 ############### Preparing for PDB output


 ############### Processing extra restraints explicitly defined

 No 'NOTE BUSTER_TARGET' cards or -target?? args found (used to read coordinates files for soft ncs to external)
 No 'NOTE BUSTER_OCCSUM'     cards found - so not using sum of occupancy restraints
 No 'NOTE BUSTER_DISTANCE'   cards found - so not setting up any additional atom-atom distance restraints
 No 'NOTE BUSTER_UTILANGLE'  cards found - so not setting up any additional restraints on any angle
 No 'NOTE BUSTER_UTILTOR'    cards found - so not setting up any additional sinusoidal restraints on torsions
 
 --- Have some similarity definitions 
     Similarity restraints on RMSD use superposition routines from: 
     Coutsias, E.A., Seok, C., Dill, K.A.(2004)
     "Using quaternions to calculate RMSD", J. Comput. Chem., 25:1849-1857 

 Have found    1 'NOTE BUSTER_SIM_DEFINE' cards 
     Maximum number of chains involved in any SIM group (excluding the template)=     1


 ########## Interpreting card: NOTE BUSTER_SIM_DEFINE ncsautoAB ncsautoABset B
     Number of atoms in template set=  2770
     Number of related chains (other than template) in SIM set=    1
     List of chains to be restrained (excluding template)=   B   
     Pairwise RMSD, XYZ in Angstroms, for SIM group: ncsautoAB
                 A         B   
     A         -----     ----- 
     B         0.563     ----- 
     Overall RMSD,XYZ for the SIM group is     0.563 Angstroms
     Pairwise rms deviation of [individual B's minus their set/chain average] in Angs**2
                 A         B         Bavg
     A         -----     -----     64.75
     B         15.03     -----     53.93
     Overall RMSD,B   for the SIM group is    15.027 Angs**2
     The number of common atoms for the above fits:
                 A         B   
     A         -----     ----- 
     B          2757     ----- 
     (compared to the template set that has    2770 atoms in it)
     List of atoms that are absent in one or more chains for this group: 
                                    A    B   
                    C1      401     +    -
                    O1      401     +    -
                    O2      401     +    -
                    C2      401     +    -
                    C3      401     +    -
                    O7      401     +    -
                    C4      401     +    -
                    C5      401     +    -
                    O3      401     +    -
                    O4      401     +    -
                    C6      401     +    -
                    O5      401     +    -
                    O6      401     +    -
     (key  +  atom present for this chain 
           -  atom absent  for this chain)
 transforms for each of the superpositions orthogonal matrix r11 r12 r13 r21 r22 r23 r31 r32 r33 tx ty tz (PDBSET syntax): 
TRANS_AB -0.878293 -0.477959 -0.012501 -0.478009  0.878354  0.001184  0.010414  0.007016 -0.999921    33.844    32.894    15.282
 reverse transforms 
TRANS_BA -0.878293 -0.478009  0.010414 -0.477959  0.878354  0.007016 -0.012501  0.001184 -0.999921    45.289   -12.824    15.665
 
 Setting up LSSR interatomic distance restraints
 Interpreting card: NOTE BUSTER_SIM_RESTRAIN_LSSR ncsautoAB 6.666
        setting up interatomic distance pair restraints with:
        Dmin=         0.00
        Dmax=         5.50
        sigma=        0.20
        Vmax=         3.00
        weight=       6.67
        number of distance pairs defined for this set is      34102
 Have not supplied either '-sim_swap_equiv' or '-sim_swap_equiv_plus' argument 
   so will not try swaping equivalent atoms to improve NCS and/or target agreement.
 
 NCS by LSSR outlier analysis
 -autoncs option automatic NCS prune routine -------- 
 Residues pruned out by gradient condition:
           A|27     A|125     A|135     A|145     A|147     A|148     A|149
          A|150     A|151     A|173     A|182     A|185     A|186     A|187
          A|188     A|189     A|211     A|212     A|215     A|216     A|217
          A|219     A|220     A|253     A|259     A|260     A|264     A|344
          A|345    A|ENDA      B|27     B|125     B|135     B|145     B|147
          B|148     B|149     B|150     B|151     B|173     B|182     B|185
          B|186     B|187     B|188     B|189     B|211     B|212     B|215
          B|216     B|217     B|219     B|220     B|253     B|259     B|260
          B|264     B|344     B|345    B|ENDA
  
 Residues pruned out by function/max condition:
          A|149!    A|187!    A|188!    A|345!   A|ENDA!    B|149!    B|187!
          B|188!    B|345!   B|ENDA!
  
 Total number of residues for which LSSR restraints have been pruned out is       60 out of      698 participating
 Number of LSSR restraints pruned out is      3699 this is   10.8% of the total           34102
  
 -autoncs .Gelly cards suitable for adaptation for manual definition/pruning
           (note auto pruning is done in a slightly different way so exact results may differ slightly)
 -autoncs option .Gelly cards also written to file  auto_gelly_cards.txt
NOTE BUSTER_SET ncsautoXcld = Water    # card added - produced by -autoncs
NOTE BUSTER_SET ncsautoXcld = ncsautoXcld + { A|27 A|125 A|135 A|145 A|147 }    # card added - produced by -autoncs
NOTE BUSTER_SET ncsautoXcld = ncsautoXcld + { A|148 A|149 A|150 A|151 A|173 }    # card added - produced by -autoncs
NOTE BUSTER_SET ncsautoXcld = ncsautoXcld + { A|182 A|185 A|186 A|187 A|188 }    # card added - produced by -autoncs
NOTE BUSTER_SET ncsautoXcld = ncsautoXcld + { A|189 A|211 A|212 A|215 A|216 }    # card added - produced by -autoncs
NOTE BUSTER_SET ncsautoXcld = ncsautoXcld + { A|217 A|219 A|220 A|253 A|259 }    # card added - produced by -autoncs
NOTE BUSTER_SET ncsautoXcld = ncsautoXcld + { A|260 A|264 A|344 A|345 A|ENDA }    # card added - produced by -autoncs
NOTE BUSTER_SET ncsautoABset = Chain_A \ ncsautoXcld    # card added - produced by -autoncs
NOTE BUSTER_SIM_DEFINE ncsautoAB ncsautoABset B    # card added - produced by -autoncs
NOTE BUSTER_SIM_RESTRAIN_LSSR ncsautoAB 6.666    # card added - produced by -autoncs

 LSSR function contribution before pruning     25794.8
                             after pruning      9233.0
 Graph of which residues pruned from LSSR: ncs_pruned.mtv
 Graph of LSSR function outliers ncs_function_outliers.mtv
 Graph of LSSR gradient outliers ncs_gradient_outliers.mtv
 LSSR - analysis of distance difference distribution for NCS
         DeltaD less than 0.5 Angs:  proportion of population= 98.83% average DeltaD of subset     0.009 Angs
 Histogram of LSSR deltaD distribution ncs_histogram.mtv
 
 Interpreting card: NOTE BUSTER_TLS_SET tls1 { A|* }
 Interpreting card: NOTE BUSTER_TLS_SET tls2 { B|* }
 Interpreting card: NOTE BUSTER_TLS_SET tls1 { A|* }
 Interpreting card: NOTE BUSTER_TLS_T   tls1     -1.79412E-01 -8.22794E-02  2.45470E-01 -1.90737E-02  4.55243E-02  1.70868E-02
 Interpreting card: NOTE BUSTER_TLS_L   tls1      1.20658E+00  7.85380E-01  6.16119E-01  1.76434E-01 -3.54449E-01  2.45657E-01
 Interpreting card: NOTE BUSTER_TLS_S   tls1      1.76213E-01 -1.34977E-01  2.10827E-01 -7.74912E-02 -1.15771E-01 -8.80908E-02 -2.18500E-02 -3.23633E-02
 Interpreting card: NOTE BUSTER_TLS_O   tls1     -2.33295E+01 -8.34950E+01  1.14606E+01
 Interpreting card: NOTE BUSTER_TLS_SET tls2 { B|* }
 Interpreting card: NOTE BUSTER_TLS_T   tls2     -2.01883E-01 -1.38784E-01  3.45547E-01  1.26053E-02  3.36128E-02 -9.22257E-03
 Interpreting card: NOTE BUSTER_TLS_L   tls2      1.25664E+00  8.56587E-01  8.72291E-01  6.16143E-01 -7.38225E-01 -6.25366E-01
 Interpreting card: NOTE BUSTER_TLS_S   tls2     -1.32166E-02  1.45295E-01 -8.73965E-02  1.30264E-01  2.20430E-01  1.24629E-02 -6.42129E-02  2.72582E-02
 Interpreting card: NOTE BUSTER_TLS_O   tls2      2.19831E+01 -7.17259E+01  3.45078E+00
 Interpreting card: NOTE BUSTER_TLS_FIX tls1 ALL
 Interpreting card: NOTE BUSTER_TLS_FIX tls2 ALL


 TLS information. Total number of groups:     2


 Group   Natoms    FixRB   FixTLS   Group Tag    Group Spec
     1     2770      Y        Y     tls1    { A|* }
     2     2767      Y        Y     tls2    { B|* }
 
NOTE BUSTER_TLS_SET tls1 { A|* }
NOTE BUSTER_TLS_T   tls1     -1.79412E-01 -8.22794E-02  2.45470E-01 -1.90737E-02  4.55243E-02  1.70868E-02
NOTE BUSTER_TLS_L   tls1      1.20658E+00  7.85380E-01  6.16119E-01  1.76434E-01 -3.54449E-01  2.45657E-01
NOTE BUSTER_TLS_S   tls1      1.76213E-01 -1.34977E-01  2.10827E-01 -7.74912E-02 -1.15771E-01 -8.80908E-02 -2.18500E-02 -3.23633E-02
NOTE BUSTER_TLS_O   tls1     -2.33295E+01 -8.34950E+01  1.14606E+01
NOTE BUSTER_TLS_FIX tls1 ALL
 
NOTE BUSTER_TLS_SET tls2 { B|* }
NOTE BUSTER_TLS_T   tls2     -2.01883E-01 -1.38784E-01  3.45547E-01  1.26053E-02  3.36128E-02 -9.22257E-03
NOTE BUSTER_TLS_L   tls2      1.25664E+00  8.56587E-01  8.72291E-01  6.16143E-01 -7.38225E-01 -6.25366E-01
NOTE BUSTER_TLS_S   tls2     -1.32166E-02  1.45295E-01 -8.73965E-02  1.30264E-01  2.20430E-01  1.24629E-02 -6.42129E-02  2.72582E-02
NOTE BUSTER_TLS_O   tls2      2.19831E+01 -7.17259E+01  3.45078E+00
NOTE BUSTER_TLS_FIX tls2 ALL


 TLS: mmCIF group specification begin


#
loop_
_pdbx_refine_tls_group.id
_pdbx_refine_tls_group.refine_tls_id
_pdbx_refine_tls_group.beg_PDB_ins_code
_pdbx_refine_tls_group.beg_auth_asym_id
_pdbx_refine_tls_group.beg_auth_seq_id
_pdbx_refine_tls_group.end_PDB_ins_code
_pdbx_refine_tls_group.end_auth_asym_id
_pdbx_refine_tls_group.end_auth_seq_id
_pdbx_refine_tls_group.selection
_pdbx_refine_tls_group.selection_details
   1   1 . A   -1 . A  346 . '{ A|* }'
   2   1 . A  401 . A  401 . '{ A|* }'
   3   2 . B   -1 . B  346 . '{ B|* }'
   4   2 . B  401 . B  401 . '{ B|* }'
#


 TLS: mmCIF group specification end


 Interpreting card: NOTE BUSTER_ANISO_SET Empty
 Interpreting card: NOTE BUSTER_ANISO_WEIGHT SCA  16.0
 Interpreting card: NOTE BUSTER_ANISO_WEIGHT SPH   0.6    0.6
 Interpreting card: NOTE BUSTER_ANISO_WEIGHT SIM   7.8    4.8    2.6    0
 Interpreting card: NOTE BUSTER_ANISO_WEIGHT RIG  21.5   21.5    3.7    0


 After TLS and ANISO: Number of atoms with fixed/combined xyz =     0
 After TLS and ANISO: Number of atoms with fixed/combined B   =     0
 After TLS and ANISO: Number of atoms with fixed/combined OCC =  6118
 After TLS and ANISO: Number of atoms with fixed/combined BIJ =  6118
 After TLS and ANISO: Number of atoms in a TLS group          =  5537
                                     cf total number of atoms =  6118
 

 ############### Setup state vector

 No free_vect_ulim_xyz argument found: using default of  100.000
 No free_vect_llim_xyz argument found: using default of -100.000
 Number of parameters added for TLS description                 =       0
 Number of parameters to describe molecular conformation        =   24472

 ############### Setup free vector

 Number of free parameters to be adjusted in refinement   =   24472

 ############### Setting up own geometry


 No command line argument '-tnt_fn' so will use gelly to do geometry function calculations

 ============= Call TNT functions


 ============= Get TNT symmetry information

 
      Getting symmetry operators from TNT. 
          gelly will classify symmetry using pdb-like convention: 
               SYMOP   SYMMETRY
              NNNMMM   OPERATOR
                1555   X,Y,Z
                2555   -Y,X-Y,Z
                3555   -X+Y,-X,Z
                4555   -X,-Y,1/2+Z
                5555   Y,-X+Y,1/2+Z
                6555   X-Y,X,1/2+Z
          where NNN -> operator number and MMM -> translation vector
 

 ============= Own Gelly geometry/restraints setup


 ----------- Identify bond-types


    Setting up GEOMBONDREC_TYPE_HASH* to be able to map a given bond type (e.g.
    "single") to an internal integer (GBOND_TYPE=1) etc.

    Using default  GEOMBONDREC_TYPE_HASH_STR='SING* 1 DOUB* 2 TRIP* 3 AROM* 4 DELO* 4 METAL* 5':
    to override default use a NOTE BUSTER_GEOMBONDREC_TYPE_HASH_STR gelly card.

 ----------- Classify torsion into peptide omega and other


 ----------- Setup of non-bonded terms

 Ideal-distance contact term setup:
    Restraint function constants (normally these values are from NOTE BUSTER_IDEAL_CONTACT_* cards)
    Sigma for general contacts      0.200 Angs
    Sigma for 1-4 contacts          0.100 Angs (contacts across a torsion)
    Sigma for 1-5 contacts          0.150 Angs
    Sigma for 1-N contacts          0.200 Angs
    Sigma for HBOND pairs           0.100 Angs (contacts between HB_types A and D or B's)
    Contact distance increment for each atom in HBOND          -0.150 Angs 
    Contact distance increment for H-bond acceptor              0.100 Angs 
    Contact distance increment for 1-4 with an O atom          -0.050 Angs 
    Contact distance increment for 1-4 with an N atom          -0.180 Angs 
    Contact distance increment for 1-4 with an C atom          -0.370 Angs 
    Contact distance increment for 1-4 with an S atom          -0.150 Angs 
    Contact distance increment for 1-4 with an P atom          -0.150 Angs 
    Contact distance increment for 1-4 with any other atom     -0.150 Angs 
    All atoms matched for CCP4 atom type.
    Number of CCP4 atom types used is         24 (H-bound and not H-bound counted separately)

 Setting up short/ideal-distance contacts
   Using gelly special position handling. (For TNT instead rerun with argument '-special_tnt')

   No command line argument '-special_all' specified. We will auto-generate
   EXCLUDE cards only for atom-atom contacts across symmetry (i.e. on special
   position) when this involves a contact within a residue to itself.

   Have set up non-bonded exclusions - 1-3 contacts
   Atom A|27:CA         makes the most 1-3 contacts:  6

   Have set up non-bonded exclusions - 1-4 contacts
   Atom A|27:N          makes the most 1-4 contacts:  7

   Have set up non-bonded exclusions - 1-5 contacts
   Atom A|28:C.A        makes the most 1-5 contacts: 10

 ############################################################################################################
 ###################################################### setting up contact terms
 ############################################################################################################


 ============= Call Gelly versions of TNT functions

 WARNING: 1-4 contact without 1-(2,3)-4 data for A|401:O2(CIT)        and A|401:C3(CIT)        X-X
 WARNING: 1-4 contact without 1-(2,3)-4 data for A|401:C2(CIT)        and A|401:O6(CIT)        X-X
 WARNING: 1-4 contact without 1-(2,3)-4 data for A|401:O7(CIT)        and A|401:O5(CIT)        X-X
 WARNING: 1-4 contact without 1-(2,3)-4 data for A|401:C4(CIT)        and A|401:O6(CIT)        X-X

       TERM      weight      number      TNT_fn    Gelly_fn        diff
       BOND       2.000        5652     781.393     781.393       0.000
       ANGL       2.000        7670    4134.921    4134.921       0.000
       IMPR       0.000           0       0.000       0.000       0.000
       TORS       2.000        1963    2832.552    2832.552       0.000
       TRIG       2.000           0       0.000       0.000       0.000
       PLAN       5.000         963    1899.535    1899.535       0.000
       CONT       5.000           0      46.470       0.000      46.470
       BCOR      10.000        5630    1882.390    3725.064   -1842.674


 ============= Call Gelly geometry functions

   Switching torsion functional form to sinusoidal rather than split harmonic functional.
      if you want this not to happen then rerun with '-torsharm'
      Weight for sinusoidal torsion=     2.000
   BCOR function/gradient will be split between H and non-H atoms (as for any atom pair). Use '-bcorrel_all_in_hydrogens' to change this.

 ----------- Fix B-correlation restraints

 
 Checking for bonds that do not have a bcorrel restraint defined
 We will wipe all existing BCORrels if OCC=0.0 atom(s) is/are involved. This can be' switched off via the '-bcorrel_nowipe_zeroocc' flag.
 We will wipe all existing BCORrels if only non-hydrogen atom(s) with fixed B(iso) are involved. This can be switched off via the '-bcorrel_nowipe_fixb_nonhydrogens' flag.
 We will keep all existing BCORrels if hydrogen atom(s) with fixed B(iso) is/are involved. This can be switched off via the '-bcorrel_wipe_fixb_hydrogens' flag.
 No -bcorrel_missing_sigma found. So will set sigma to     2.5 Ang**2 for any bond with no existing BCORrel
 No -bcorrel_missing_sigma_hydrogens found. So will set sigma to     1.0 Ang**2 for any bond with no existing BCORrel involving hydrogens

 No active BCORels found involving OCC=0.0 atom(s)


 No active BCORels found involving fixed-B (only) non-hydrogen atom(s)

    add a default BCOR for bond A|401:C1(CIT) A|401:C2(CIT)
    add a default BCOR for bond A|401:C1(CIT) A|401:O1(CIT)
    add a default BCOR for bond A|401:C1(CIT) A|401:O2(CIT)
    add a default BCOR for bond A|401:C2(CIT) A|401:C3(CIT)
    add a default BCOR for bond A|401:C3(CIT) A|401:C4(CIT)
    add a default BCOR for bond A|401:C3(CIT) A|401:C6(CIT)
    add a default BCOR for bond A|401:C3(CIT) A|401:O7(CIT)
    add a default BCOR for bond A|401:C4(CIT) A|401:C5(CIT)
    add a default BCOR for bond A|401:C5(CIT) A|401:O3(CIT)
    add a default BCOR for bond A|401:C5(CIT) A|401:O4(CIT)
    add a default BCOR for bond A|401:C6(CIT) A|401:O5(CIT)
    add a default BCOR for bond A|401:C6(CIT) A|401:O6(CIT)
    add a default BCOR for bond B|401:C01(LIG) B|401:N02(LIG)
    add a default BCOR for bond B|401:N02(LIG) B|401:C03(LIG)
    add a default BCOR for bond B|401:C03(LIG) B|401:C04(LIG)
    add a default BCOR for bond B|401:C03(LIG) B|401:C05(LIG)
    add a default BCOR for bond B|401:C05(LIG) B|401:C06(LIG)
    add a default BCOR for bond B|401:C05(LIG) B|401:C10(LIG)
    add a default BCOR for bond B|401:C06(LIG) B|401:N07(LIG)
    add a default BCOR for bond B|401:N07(LIG) B|401:N08(LIG)
    add a default BCOR for bond B|401:N08(LIG) B|401:C09(LIG)
    add a default BCOR for bond B|401:N08(LIG) B|401:C10(LIG)

 Have added a total of        22 BCORrels for bonds which do not already have a BCORrel


 No bonds (for potential BCORrel addition) were skipped due to both atoms having constant/fixed B(iso)


 ----------- Deactivate hydrogen-free planes if hydrogen atoms present

 A total of       0 planes have been deactivated because they are duplicated hydrogen-free and with-hydrogen forms.

 ----------- Handle peptide omega angle(s)

      Torsion angles split into peptide omega and other
         Number of peptide omega angles is       698
         Number of other torsion is             1265
      Peptide omega angles are monitored rather than directly restrained (PLANE used instead).

 ----------- Fix cis-PRO

 Now apply the fix to get correct restraints for cis PRO residues
   Have got      11 bonds records and      26 angles
   from GEOMETRY cards for the special PROC residue
   Weight for ideal-distance contact term=     4.000

 ----------- Prepare SSBOND/LINK for output PDB file


 ----------- Prepare for QM


 ----------- Prepare symmetry in utility distance restraints


 ############### Setting up Rhofit contacts (if needed)

 No ligand-(fixed protein) contact term will be used

 ############### Working out initial function and gradient


   MaxLik Scaling Cycle 0002
 
 Initial functional value = 5.708086E+05 rms gradient=      57.6748
 X-ray contribution to function value = 5.405172E+05
 Geometry contribution (inc ncs)      =      30291.4
 Geometry function summary
     term                           number     weight       rms        GooF      function     fn/numb
     ================================================================================================
     BOND bond lengths (angs)       5652       2.000      0.0053       0.263       781.39       0.138
     ANGL bond angles (degs)        7670       2.000        0.87       0.519      4134.92       0.539
     TORS split-harmonic tors       1265       0.000       16.80       1.058         0.00       0.000
     SINTOR sinusoidal tors         1265       2.000       16.80                  2157.79       1.706
     PLAN general plane (angs)       963       5.000      0.0126       0.628      1899.54       1.973
     BCORrelations (angs^2)         5652      10.000       0.720       0.257      3731.08       0.660
     CONT (bad contacts)               0       5.000                                 0.00
     IDEAL(ideal-dist contact)      5071       4.000                              8314.48
     CHIRAL (gelly semiharmon)       740       5.000                                39.21
     SIM similarity restraint for NCS and/or TARGET                               9232.97
     OCCSUM restraint on sum of occupanies                                           0.00       as none defined
     DISTAN utility restraints interatomic distance                                  0.00       as none defined
     UTILANGLE utility angle restraints                                              0.00       as none defined
     UTILTOR utility restraints on torsion angles                                    0.00       as none defined
     Peptide omega torsion angles                           2.67 degs - monitor only
 
 WARNING: Have |delta/sigma| deviations > 5.0 sigma. Number of outliers for each term: 
 WARNING:         4 bond angles.  Worst is     5.1 sigs   134.44 degs A|28:C.B(ASN)=A|29:N(ASN)=A|29:CA(ASN)

 Weighted rms      0.0053         0.871  (temporary rms bond and angle for BUSTER to pick up!)
 Weighted intra-residual rms(bond) for StdProtein (excluding H)      0.0051
 Weighted intra-residual rms(bond) for Buffer     (excluding H)      0.0037

 ############### Running initial check


 Initial Geometry Sanity Check

 Check for duplicate bonds: no duplicates found
 
 Check for duplicate or missing bond angles: all are OK
 Check for bond between an atom and itself not involving symmetry:  all are OK
   There are no bad contacts where Delta/sigma is greater than     6.5
   Worst contact dist=   2.831 std=   3.185 sigma=   0.107 abs(Delta/sigma)=    3.311 ats A|28:CB.B (ASN) A|29:N (ASN)
   It is likely that the geometry libraries, sequence info and links match the input molecule
 GEOMETRY CHECK OVERALL:  PASS


 ############### Creating geometry/screen report


 ############### Option "tao" - run Tao optimizer

 TAO: Toolkit for Advanced Optimization. Reference: 
     Munson, T., Sarich, J., Wild, S., Benson, S., McInnes, L.C. (2018)
     "TAO User Manual (Revision 3.9)", Mathematics and Computer Science
     Division, Argonne National Laboratory
     ANL/MCS-TM-322, http://www.mcs.anl.gov/petsc/
 
 Have found -keeppdb flag so will write out a .pdb file every 1000000 its during opt
 Will apply TAO optimization 
 Maximum number of iterations      100
 Convergence test on Grms       4.0000 (-glim)
 Limit on rms displacement to original position (rmsD2init): NONE test inactive (-dlim)

    analysing argument no 0 : -autoncs with j=0
    analysing argument no 1 : -hydrogens_input_occ2zero with j=0
    analysing argument no 2 : -hydrogens_output_occ2heavy with j=0
    analysing argument no 3 : -screen with j=0
    analysing argument no 4 : 100 with j=0
    analysing argument no 5 : -screen_sigma with j=0
    analysing argument no 6 : 3.0 with j=0
    analysing argument no 7 : -keeppdb with j=0
    analysing argument no 8 : 1000000 with j=0
    analysing argument no 9 : -taomonitor_verbose with j=0
    found argument starting with "-tao" : -taomonitor_verbose
    analysing argument no 10 : 2 with j=1
    found value after -tao/-options_/-viewer_ argument : "2"
 PETSC/TAO: setting option "-taomonitor_verbose" = "2"


   MaxLik Scaling Cycle 0003
   Iters     Tot_Funct    Xray_Funct    Geom_Funct          Grms     rmsD2init     maxD2init             R         Rfree           LLG       LLGfree     rms(bond)    rms(angle)       Opttag_H
   Iters             K   B_IMPF_FRAG        K_MISS   B_TEMP_MISS   K_IMPF_MISS   B_IMPF_MISS        K_SOLV   B_TEMP_SOLV   K_IMPF_SOLV   B_IMPF_SOLV        B_TEMP     B_TEMP_11     B_TEMP_12     B_TEMP_22     B_TEMP_13     B_TEMP_23     B_TEMP_33      K_WILSON      B_WILSON      FGCYCLES       Scatag_H
   Iters          BOND         ANGLE      IMPROPER       TORSION    SINTORSION      TRIGONAL        PLANAR  BCORRELATION       CONTACT     IDEALDIST        CHIRAL    SIMILARITY       OCCSSUM  UTILDISTANCE     UTILANGLE   UTILTORSION        RHOFIT        ADP-01        ADP-02        ADP-03        ADP-04        ADP-05        ADP-06        ADP-07        ADP-08        ADP-09        ADP-10      RHOFITPC            QM       Funtag_H
       0  5.708086E+05  5.405172E+05       30291.4       57.6748  0.000000E+00  0.000000E+00      0.220167      0.239865  0.000000E+00  0.000000E+00    0.00527576      0.871070       Opttag_D
       0       51.8178       2.74399       1.00000  0.000000E+00       1.00000  0.000000E+00      0.835279       52.3247       1.00000       22.0555  0.000000E+00      0.478326  0.000000E+00      0.478326  0.000000E+00  0.000000E+00     -0.956653       52.0422       1.49295        2       Scatag_D
       0       781.393       4134.92  0.000000E+00  0.000000E+00       2157.79  0.000000E+00       1899.54       3731.08  0.000000E+00       8314.48       39.2067       9232.97 -1.000000E+10 -1.000000E+10 -1.000000E+10 -1.000000E+10 -1.000000E+10  0.000000E+00  0.000000E+00  0.000000E+00  0.000000E+00  0.000000E+00  0.000000E+00  0.000000E+00  0.000000E+00  0.000000E+00  0.000000E+00  0.000000E+00  0.000000E+00       Funtag_D
   Geometry Restraint Deviations SCREEN Information

 

 An atom has moved more than     1.50 A from the initial/last
 position - if this was an accepted trial point, probably need to update the
 non-bonded contact list.


   MaxLik Scaling Cycle 0004

   MaxLik Scaling Cycle 0005

   MaxLik Scaling Cycle 0006

   Wilson plot

   Statistics in resolution bins after refinement cycle 0001
```


|  |  |
| --- | --- |
|  | - Rxpct factors plot- Rxpct factors table- <Log-Lik Gain>- Ln Sigma A- Correlation Coefficients |


```
   Iters     Tot_Funct    Xray_Funct    Geom_Funct          Grms     rmsD2init     maxD2init             R         Rfree           LLG       LLGfree     rms(bond)    rms(angle)
   Iters             K   B_IMPF_FRAG        K_MISS   B_TEMP_MISS   K_IMPF_MISS   B_IMPF_MISS        K_SOLV   B_TEMP_SOLV   K_IMPF_SOLV   B_IMPF_SOLV        B_TEMP     B_TEMP_11     B_TEMP_12     B_TEMP_22     B_TEMP_13     B_TEMP_23     B_TEMP_33      K_WILSON      B_WILSON      FGCYCLES
   Iters          BOND         ANGLE      IMPROPER       TORSION    SINTORSION      TRIGONAL        PLANAR  BCORRELATION       CONTACT     IDEALDIST        CHIRAL    SIMILARITY       OCCSSUM  UTILDISTANCE     UTILANGLE   UTILTORSION        RHOFIT        ADP-01        ADP-02        ADP-03        ADP-04        ADP-05        ADP-06        ADP-07        ADP-08        ADP-09        ADP-10      RHOFITPC            QM
       1  5.701843E+05  5.397781E+05       30406.2       51.5384  7.746514E-04    0.00660064      0.219786      0.239779    0.00167211  2.391370E-04    0.00544310      0.872428       Opttag_D
       1       51.7899       2.70865       1.00000  0.000000E+00       1.00000  0.000000E+00      0.835081       52.1641       1.00000       22.1495  0.000000E+00      0.466619  0.000000E+00      0.466619  0.000000E+00  0.000000E+00     -0.933238       52.0422       1.49295        3       Scatag_D
       1       831.749       4147.82  0.000000E+00  0.000000E+00       2156.77  0.000000E+00       1916.07       3760.66  0.000000E+00       8314.76       39.6058       9238.76 -1.000000E+10 -1.000000E+10 -1.000000E+10 -1.000000E+10 -1.000000E+10  0.000000E+00  0.000000E+00  0.000000E+00  0.000000E+00  0.000000E+00  0.000000E+00  0.000000E+00  0.000000E+00  0.000000E+00  0.000000E+00  0.000000E+00  0.000000E+00       Funtag_D
   Geometry Restraint Deviations SCREEN Information

 

   MaxLik Scaling Cycle 0007

   Wilson plot

   Statistics in resolution bins after refinement cycle 0002
```


|  |  |
| --- | --- |
|  | - Rxpct factors plot- Rxpct factors table- <Log-Lik Gain>- Ln Sigma A- Correlation Coefficients |


```
   Iters     Tot_Funct    Xray_Funct    Geom_Funct          Grms     rmsD2init     maxD2init             R         Rfree           LLG       LLGfree     rms(bond)    rms(angle)
   Iters             K   B_IMPF_FRAG        K_MISS   B_TEMP_MISS   K_IMPF_MISS   B_IMPF_MISS        K_SOLV   B_TEMP_SOLV   K_IMPF_SOLV   B_IMPF_SOLV        B_TEMP     B_TEMP_11     B_TEMP_12     B_TEMP_22     B_TEMP_13     B_TEMP_23     B_TEMP_33      K_WILSON      B_WILSON      FGCYCLES
   Iters          BOND         ANGLE      IMPROPER       TORSION    SINTORSION      TRIGONAL        PLANAR  BCORRELATION       CONTACT     IDEALDIST        CHIRAL    SIMILARITY       OCCSSUM  UTILDISTANCE     UTILANGLE   UTILTORSION        RHOFIT        ADP-01        ADP-02        ADP-03        ADP-04        ADP-05        ADP-06        ADP-07        ADP-08        ADP-09        ADP-10      RHOFITPC            QM
       2  5.696491E+05  5.390471E+05       30602.0       39.9678    0.00169655    0.00994958      0.219367      0.239670    0.00332642  5.030045E-04    0.00569694      0.873905       Opttag_D
       2       51.7611       2.67494       1.00000  0.000000E+00       1.00000  0.000000E+00      0.834909       52.0489       1.00000       22.2025  0.000000E+00      0.455190  0.000000E+00      0.455190  0.000000E+00  0.000000E+00     -0.910380       52.0422       1.49295        1       Scatag_D
       2       911.135       4161.88  0.000000E+00  0.000000E+00       2155.58  0.000000E+00       1942.25       3816.44  0.000000E+00       8320.73       40.3522       9253.62 -1.000000E+10 -1.000000E+10 -1.000000E+10 -1.000000E+10 -1.000000E+10  0.000000E+00  0.000000E+00  0.000000E+00  0.000000E+00  0.000000E+00  0.000000E+00  0.000000E+00  0.000000E+00  0.000000E+00  0.000000E+00  0.000000E+00  0.000000E+00       Funtag_D
   Geometry Restraint Deviations SCREEN Information

 
       3  5.690809E+05  5.376278E+05       31453.1       48.0387    0.00379776     0.0213107      0.218515      0.239435    0.00653849  9.613792E-04    0.00629831      0.883191       Opttag_D
       3       51.7042       2.61148       1.00000  0.000000E+00       1.00000  0.000000E+00      0.834595       51.8709       1.00000       22.2715  0.000000E+00      0.434076  0.000000E+00      0.434076  0.000000E+00  0.000000E+00     -0.868152       52.0422       1.49295        1       Scatag_D
       3       1113.65       4250.80  0.000000E+00  0.000000E+00       2153.14  0.000000E+00       2011.11       4229.14  0.000000E+00       8345.21       41.7870       9308.30 -1.000000E+10 -1.000000E+10 -1.000000E+10 -1.000000E+10 -1.000000E+10  0.000000E+00  0.000000E+00  0.000000E+00  0.000000E+00  0.000000E+00  0.000000E+00  0.000000E+00  0.000000E+00  0.000000E+00  0.000000E+00  0.000000E+00  0.000000E+00       Funtag_D
       4  5.688468E+05  5.363347E+05       32512.2       71.8865    0.00597597     0.0343963      0.217717      0.239191    0.00946479    0.00145254    0.00684759      0.902193       Opttag_D
       4       51.6524       2.55583       1.00000  0.000000E+00       1.00000  0.000000E+00      0.834330       51.7499       1.00000       22.3050  0.000000E+00      0.415865  0.000000E+00      0.415865  0.000000E+00  0.000000E+00     -0.831730       52.0422       1.49295        1       Scatag_D
       4       1316.36       4435.68  0.000000E+00  0.000000E+00       2150.72  0.000000E+00       2095.12       4701.93  0.000000E+00       8384.41       42.5789       9385.36 -1.000000E+10 -1.000000E+10 -1.000000E+10 -1.000000E+10 -1.000000E+10  0.000000E+00  0.000000E+00  0.000000E+00  0.000000E+00  0.000000E+00  0.000000E+00  0.000000E+00  0.000000E+00  0.000000E+00  0.000000E+00  0.000000E+00  0.000000E+00       Funtag_D
       5  5.685160E+05  5.363112E+05       32204.8       36.9773    0.00611418     0.0361258      0.217691      0.239155    0.00951798    0.00155640    0.00673188      0.888139       Opttag_D
       5       51.6498       2.55497       1.00000  0.000000E+00       1.00000  0.000000E+00      0.834327       51.7629       1.00000       22.2958  0.000000E+00      0.416401  0.000000E+00      0.416401  0.000000E+00  0.000000E+00     -0.832801       52.0422       1.49295        1       Scatag_D
       5       1272.25       4298.56  0.000000E+00  0.000000E+00       2150.51  0.000000E+00       2103.40       4568.09  0.000000E+00       8389.22       42.0535       9380.74 -1.000000E+10 -1.000000E+10 -1.000000E+10 -1.000000E+10 -1.000000E+10  0.000000E+00  0.000000E+00  0.000000E+00  0.000000E+00  0.000000E+00  0.000000E+00  0.000000E+00  0.000000E+00  0.000000E+00  0.000000E+00  0.000000E+00  0.000000E+00       Funtag_D
       6  5.681906E+05  5.358995E+05       32291.1       33.1449    0.00713689     0.0442582      0.217411      0.239008     0.0104498    0.00191080    0.00673432      0.891027       Opttag_D
       6       51.6307       2.53806       1.00000  0.000000E+00       1.00000  0.000000E+00      0.834248       51.7640       1.00000       22.2824  0.000000E+00      0.412078  0.000000E+00      0.412078  0.000000E+00  0.000000E+00     -0.824156       52.0422       1.49295        1       Scatag_D
       6       1273.17       4326.56  0.000000E+00  0.000000E+00       2149.37  0.000000E+00       2148.75       4537.66  0.000000E+00       8414.51       41.4952       9399.57 -1.000000E+10 -1.000000E+10 -1.000000E+10 -1.000000E+10 -1.000000E+10  0.000000E+00  0.000000E+00  0.000000E+00  0.000000E+00  0.000000E+00  0.000000E+00  0.000000E+00  0.000000E+00  0.000000E+00  0.000000E+00  0.000000E+00  0.000000E+00       Funtag_D
       7  5.678980E+05  5.352232E+05       32674.8       38.8582    0.00877594     0.0567134      0.216965      0.238798     0.0119802    0.00238447    0.00683950      0.898405       Opttag_D
       7       51.6030       2.51069       1.00000  0.000000E+00       1.00000  0.000000E+00      0.834117       51.7560       1.00000       22.2704  0.000000E+00      0.403332  0.000000E+00      0.403332  0.000000E+00  0.000000E+00     -0.806664       52.0422       1.49295        1       Scatag_D
       7       1313.25       4398.51  0.000000E+00  0.000000E+00       2147.62  0.000000E+00       2219.64       4662.70  0.000000E+00       8455.27       41.7534       9436.02 -1.000000E+10 -1.000000E+10 -1.000000E+10 -1.000000E+10 -1.000000E+10  0.000000E+00  0.000000E+00  0.000000E+00  0.000000E+00  0.000000E+00  0.000000E+00  0.000000E+00  0.000000E+00  0.000000E+00  0.000000E+00  0.000000E+00  0.000000E+00       Funtag_D
       8  5.675723E+05  5.337668E+05       33805.4       80.5118     0.0127575     0.0873812      0.216017      0.238329     0.0152761    0.00342625    0.00712452      0.932917       Opttag_D
       8       51.5508       2.45383       1.00000  0.000000E+00       1.00000  0.000000E+00      0.833839       51.7616       1.00000       22.2469  0.000000E+00      0.382926  0.000000E+00      0.382926  0.000000E+00  0.000000E+00     -0.765852       52.0422       1.49295        1       Scatag_D
       8       1424.99       4742.94  0.000000E+00  0.000000E+00       2143.54  0.000000E+00       2387.68       4986.75  0.000000E+00       8563.23       44.4581       9511.86 -1.000000E+10 -1.000000E+10 -1.000000E+10 -1.000000E+10 -1.000000E+10  0.000000E+00  0.000000E+00  0.000000E+00  0.000000E+00  0.000000E+00  0.000000E+00  0.000000E+00  0.000000E+00  0.000000E+00  0.000000E+00  0.000000E+00  0.000000E+00       Funtag_D
       9  5.671415E+05  5.329305E+05       34211.0       33.3503     0.0151720      0.105742      0.215476      0.238084     0.0171688    0.00393294    0.00723188      0.920420       Opttag_D
       9       51.5240       2.42211       1.00000  0.000000E+00       1.00000  0.000000E+00      0.833681       51.7710       1.00000       22.2349  0.000000E+00      0.370087  0.000000E+00      0.370087  0.000000E+00  0.000000E+00     -0.740173       52.0422       1.49295        1       Scatag_D
       9       1468.26       4616.72  0.000000E+00  0.000000E+00       2141.38  0.000000E+00       2491.26       5254.46  0.000000E+00       8632.91       46.5544       9559.50 -1.000000E+10 -1.000000E+10 -1.000000E+10 -1.000000E+10 -1.000000E+10  0.000000E+00  0.000000E+00  0.000000E+00  0.000000E+00  0.000000E+00  0.000000E+00  0.000000E+00  0.000000E+00  0.000000E+00  0.000000E+00  0.000000E+00  0.000000E+00       Funtag_D
      10  5.669348E+05  5.327294E+05       34205.3       26.6251     0.0158472      0.111422      0.215361      0.238008     0.0176238    0.00410248    0.00722795      0.920773       Opttag_D
      10       51.5203       2.41446       1.00000  0.000000E+00       1.00000  0.000000E+00      0.833624       51.7677       1.00000       22.2471  0.000000E+00      0.366744  0.000000E+00      0.366744  0.000000E+00  0.000000E+00     -0.733489       52.0422       1.49295        1       Scatag_D
      10       1466.67       4620.26  0.000000E+00  0.000000E+00       2140.80  0.000000E+00       2508.23       5218.45  0.000000E+00       8652.27       47.7068       9550.97 -1.000000E+10 -1.000000E+10 -1.000000E+10 -1.000000E+10 -1.000000E+10  0.000000E+00  0.000000E+00  0.000000E+00  0.000000E+00  0.000000E+00  0.000000E+00  0.000000E+00  0.000000E+00  0.000000E+00  0.000000E+00  0.000000E+00  0.000000E+00       Funtag_D
      11  5.666075E+05  5.321032E+05       34504.2       32.0567     0.0181438      0.129715      0.214980      0.237764     0.0190410    0.00461653    0.00728667      0.927398       Opttag_D
      11       51.5074       2.39102       1.00000  0.000000E+00       1.00000  0.000000E+00      0.833452       51.7711       1.00000       22.2723  0.000000E+00      0.356334  0.000000E+00      0.356334  0.000000E+00  0.000000E+00     -0.712668       52.0422       1.49295        1       Scatag_D
      11       1490.59       4686.98  0.000000E+00  0.000000E+00       2139.01  0.000000E+00       2570.23       5301.15  0.000000E+00       8720.72       50.6754       9544.88 -1.000000E+10 -1.000000E+10 -1.000000E+10 -1.000000E+10 -1.000000E+10  0.000000E+00  0.000000E+00  0.000000E+00  0.000000E+00  0.000000E+00  0.000000E+00  0.000000E+00  0.000000E+00  0.000000E+00  0.000000E+00  0.000000E+00  0.000000E+00       Funtag_D
      12  5.663658E+05  5.312890E+05       35076.8       59.9962     0.0216815      0.159136      0.214473      0.237442     0.0208837    0.00533728    0.00739898      0.948145       Opttag_D
      12       51.4888       2.36087       1.00000  0.000000E+00       1.00000  0.000000E+00      0.833202       51.8000       1.00000       22.2946  0.000000E+00      0.343529  0.000000E+00      0.343529  0.000000E+00  0.000000E+00     -0.687058       52.0422       1.49295        1       Scatag_D
      12       1536.89       4899.04  0.000000E+00  0.000000E+00       2136.94  0.000000E+00       2657.96       5406.46  0.000000E+00       8833.65       52.9310       9552.93 -1.000000E+10 -1.000000E+10 -1.000000E+10 -1.000000E+10 -1.000000E+10  0.000000E+00  0.000000E+00  0.000000E+00  0.000000E+00  0.000000E+00  0.000000E+00  0.000000E+00  0.000000E+00  0.000000E+00  0.000000E+00  0.000000E+00  0.000000E+00       Funtag_D
      13  5.660655E+05  5.308187E+05       35246.8       30.7508     0.0240499      0.179339      0.214177      0.237245     0.0219479    0.00577403    0.00740590      0.943712       Opttag_D
      13       51.4790       2.34381       1.00000  0.000000E+00       1.00000  0.000000E+00      0.833044       51.8290       1.00000       22.3058  0.000000E+00      0.336021  0.000000E+00      0.336021  0.000000E+00  0.000000E+00     -0.672043       52.0422       1.49295        1       Scatag_D
      13       1539.77       4853.33  0.000000E+00  0.000000E+00       2135.67  0.000000E+00       2711.28       5479.26  0.000000E+00       8912.57       54.2933       9560.60 -1.000000E+10 -1.000000E+10 -1.000000E+10 -1.000000E+10 -1.000000E+10  0.000000E+00  0.000000E+00  0.000000E+00  0.000000E+00  0.000000E+00  0.000000E+00  0.000000E+00  0.000000E+00  0.000000E+00  0.000000E+00  0.000000E+00  0.000000E+00       Funtag_D
      14  5.658604E+05  5.305129E+05       35347.5       23.7995     0.0258833      0.195149      0.213979      0.237110     0.0226400    0.00611795    0.00738988      0.947537       Opttag_D
      14       51.4719       2.33266       1.00000  0.000000E+00       1.00000  0.000000E+00      0.832914       51.8583       1.00000       22.3108  0.000000E+00      0.331847  0.000000E+00      0.331847  0.000000E+00  0.000000E+00     -0.663694       52.0422       1.49295        1       Scatag_D
      14       1533.11       4892.76  0.000000E+00  0.000000E+00       2134.92  0.000000E+00       2739.81       5450.24  0.000000E+00       8977.14       54.3843       9565.10 -1.000000E+10 -1.000000E+10 -1.000000E+10 -1.000000E+10 -1.000000E+10  0.000000E+00  0.000000E+00  0.000000E+00  0.000000E+00  0.000000E+00  0.000000E+00  0.000000E+00  0.000000E+00  0.000000E+00  0.000000E+00  0.000000E+00  0.000000E+00       Funtag_D
      15  5.656664E+05  5.301312E+05       35535.2       30.1410     0.0283453      0.216149      0.213724      0.236959     0.0235038    0.00653296    0.00737521      0.955083       Opttag_D
      15       51.4649       2.31893       1.00000  0.000000E+00       1.00000  0.000000E+00      0.832735       51.8997       1.00000       22.3175  0.000000E+00      0.326914  0.000000E+00      0.326914  0.000000E+00  0.000000E+00     -0.653829       52.0422       1.49295        1       Scatag_D
      15       1527.03       4970.99  0.000000E+00  0.000000E+00       2134.02  0.000000E+00       2769.61       5436.42  0.000000E+00       9067.48       54.8343       9574.78 -1.000000E+10 -1.000000E+10 -1.000000E+10 -1.000000E+10 -1.000000E+10  0.000000E+00  0.000000E+00  0.000000E+00  0.000000E+00  0.000000E+00  0.000000E+00  0.000000E+00  0.000000E+00  0.000000E+00  0.000000E+00  0.000000E+00  0.000000E+00       Funtag_D
      16  5.654683E+05  5.295445E+05       35923.8       42.1096     0.0322160      0.248329      0.213334      0.236771     0.0248315    0.00706196    0.00739594      0.968417       Opttag_D
      16       51.4607       2.29866       1.00000  0.000000E+00       1.00000  0.000000E+00      0.832462       51.9634       1.00000       22.3324  0.000000E+00      0.318729  0.000000E+00      0.318729  0.000000E+00  0.000000E+00     -0.637457       52.0422       1.49295        1       Scatag_D
      16       1535.63       5110.77  0.000000E+00  0.000000E+00       2132.85  0.000000E+00       2807.03       5470.09  0.000000E+00       9211.08       56.8176       9599.49 -1.000000E+10 -1.000000E+10 -1.000000E+10 -1.000000E+10 -1.000000E+10  0.000000E+00  0.000000E+00  0.000000E+00  0.000000E+00  0.000000E+00  0.000000E+00  0.000000E+00  0.000000E+00  0.000000E+00  0.000000E+00  0.000000E+00  0.000000E+00       Funtag_D
      17  5.652667E+05  5.292852E+05       35981.5       31.1336     0.0338839      0.261917      0.213161      0.236702     0.0254184    0.00723634    0.00739420      0.968513       Opttag_D
      17       51.4648       2.29020       1.00000  0.000000E+00       1.00000  0.000000E+00      0.832330       51.9867       1.00000       22.3447  0.000000E+00      0.314693  0.000000E+00      0.314693  0.000000E+00  0.000000E+00     -0.629386       52.0422       1.49295        1       Scatag_D
      17       1534.91       5111.78  0.000000E+00  0.000000E+00       2132.48  0.000000E+00       2805.24       5455.05  0.000000E+00       9274.77       57.9807       9609.26 -1.000000E+10 -1.000000E+10 -1.000000E+10 -1.000000E+10 -1.000000E+10  0.000000E+00  0.000000E+00  0.000000E+00  0.000000E+00  0.000000E+00  0.000000E+00  0.000000E+00  0.000000E+00  0.000000E+00  0.000000E+00  0.000000E+00  0.000000E+00       Funtag_D
      18  5.650854E+05  5.290006E+05       36084.8       22.1891     0.0355807      0.275336      0.212975      0.236624     0.0260624    0.00737023    0.00741507      0.970075       Opttag_D
      18       51.4732       2.28141       1.00000  0.000000E+00       1.00000  0.000000E+00      0.832196       52.0090       1.00000       22.3605  0.000000E+00      0.309515  0.000000E+00      0.309515  0.000000E+00  0.000000E+00     -0.619030       52.0422       1.49295        1       Scatag_D
      18       1543.59       5128.28  0.000000E+00  0.000000E+00       2132.03  0.000000E+00       2802.02       5460.55  0.000000E+00       9338.37       59.4584       9620.50 -1.000000E+10 -1.000000E+10 -1.000000E+10 -1.000000E+10 -1.000000E+10  0.000000E+00  0.000000E+00  0.000000E+00  0.000000E+00  0.000000E+00  0.000000E+00  0.000000E+00  0.000000E+00  0.000000E+00  0.000000E+00  0.000000E+00  0.000000E+00       Funtag_D
      19  5.649333E+05  5.286815E+05       36251.7       24.3751     0.0375617      0.290549      0.212757      0.236526     0.0267846    0.00751194    0.00745300      0.974287       Opttag_D
      19       51.4870       2.27219       1.00000  0.000000E+00       1.00000  0.000000E+00      0.832047       52.0411       1.00000       22.3780  0.000000E+00      0.302785  0.000000E+00      0.302785  0.000000E+00  0.000000E+00     -0.605571       52.0422       1.49295        1       Scatag_D
      19       1559.42       5172.91  0.000000E+00  0.000000E+00       2131.73  0.000000E+00       2798.54       5483.03  0.000000E+00       9410.34       60.7069       9635.07 -1.000000E+10 -1.000000E+10 -1.000000E+10 -1.000000E+10 -1.000000E+10  0.000000E+00  0.000000E+00  0.000000E+00  0.000000E+00  0.000000E+00  0.000000E+00  0.000000E+00  0.000000E+00  0.000000E+00  0.000000E+00  0.000000E+00  0.000000E+00       Funtag_D
      20  5.648004E+05  5.281085E+05       36691.8       45.7266     0.0417477      0.321567      0.212347      0.236339     0.0280813    0.00777075    0.00754878      0.987588       Opttag_D
      20       51.5216       2.25704       1.00000  0.000000E+00       1.00000  0.000000E+00      0.831759       52.1294       1.00000       22.4043  0.000000E+00      0.289435  0.000000E+00      0.289435  0.000000E+00  0.000000E+00     -0.578869       52.0422       1.49295        1       Scatag_D
      20       1599.76       5315.11  0.000000E+00  0.000000E+00       2131.76  0.000000E+00       2798.51       5546.06  0.000000E+00       9564.68       62.3354       9673.62 -1.000000E+10 -1.000000E+10 -1.000000E+10 -1.000000E+10 -1.000000E+10  0.000000E+00  0.000000E+00  0.000000E+00  0.000000E+00  0.000000E+00  0.000000E+00  0.000000E+00  0.000000E+00  0.000000E+00  0.000000E+00  0.000000E+00  0.000000E+00       Funtag_D
      21  5.646146E+05  5.279833E+05       36631.3       21.7659     0.0428771      0.329513      0.212246      0.236277     0.0283648    0.00784505    0.00752008      0.983261       Opttag_D
      21       51.5356       2.25456       1.00000  0.000000E+00       1.00000  0.000000E+00      0.831680       52.1656       1.00000       22.4078  0.000000E+00      0.285762  0.000000E+00      0.285762  0.000000E+00  0.000000E+00     -0.571523       52.0422       1.49295        1       Scatag_D
      21       1587.61       5268.64  0.000000E+00  0.000000E+00       2131.91  0.000000E+00       2796.63       5500.85  0.000000E+00       9603.49       62.0747       9680.09 -1.000000E+10 -1.000000E+10 -1.000000E+10 -1.000000E+10 -1.000000E+10  0.000000E+00  0.000000E+00  0.000000E+00  0.000000E+00  0.000000E+00  0.000000E+00  0.000000E+00  0.000000E+00  0.000000E+00  0.000000E+00  0.000000E+00  0.000000E+00       Funtag_D
      22  5.644967E+05  5.278997E+05       36597.0       19.1524     0.0438431      0.336104      0.212167      0.236224     0.0285539    0.00794225    0.00748201      0.984205       Opttag_D
      22       51.5498       2.25355       1.00000  0.000000E+00       1.00000  0.000000E+00      0.831616       52.2040       1.00000       22.4073  0.000000E+00      0.283023  0.000000E+00      0.283023  0.000000E+00  0.000000E+00     -0.566045       52.0422       1.49295        1       Scatag_D
      22       1571.58       5278.76  0.000000E+00  0.000000E+00       2132.16  0.000000E+00       2796.06       5437.71  0.000000E+00       9635.38       61.6174       9683.69 -1.000000E+10 -1.000000E+10 -1.000000E+10 -1.000000E+10 -1.000000E+10  0.000000E+00  0.000000E+00  0.000000E+00  0.000000E+00  0.000000E+00  0.000000E+00  0.000000E+00  0.000000E+00  0.000000E+00  0.000000E+00  0.000000E+00  0.000000E+00       Funtag_D
      23  5.643631E+05  5.276892E+05       36673.9       24.2729     0.0461336      0.351713      0.211980      0.236117     0.0290302    0.00811582    0.00744890      0.987818       Opttag_D
      23       51.5817       2.25042       1.00000  0.000000E+00       1.00000  0.000000E+00      0.831472       52.2852       1.00000       22.4105  0.000000E+00      0.276526  0.000000E+00      0.276526  0.000000E+00  0.000000E+00     -0.553053       52.0422       1.49295        1       Scatag_D
      23       1557.70       5317.59  0.000000E+00  0.000000E+00       2132.73  0.000000E+00       2799.71       5392.32  0.000000E+00       9713.54       61.9516       9698.39 -1.000000E+10 -1.000000E+10 -1.000000E+10 -1.000000E+10 -1.000000E+10  0.000000E+00  0.000000E+00  0.000000E+00  0.000000E+00  0.000000E+00  0.000000E+00  0.000000E+00  0.000000E+00  0.000000E+00  0.000000E+00  0.000000E+00  0.000000E+00       Funtag_D
      24  5.642677E+05  5.273552E+05       36912.5       39.2444     0.0500598      0.377741      0.211682      0.235959     0.0297862    0.00835921    0.00743144      0.996419       Opttag_D
      24       51.6414       2.24668       1.00000  0.000000E+00       1.00000  0.000000E+00      0.831245       52.4290       1.00000       22.4155  0.000000E+00      0.265014  0.000000E+00      0.265014  0.000000E+00  0.000000E+00     -0.530028       52.0422       1.49295        1       Scatag_D
      24       1550.41       5410.59  0.000000E+00  0.000000E+00       2134.25  0.000000E+00       2813.39       5365.82  0.000000E+00       9844.82       63.4371       9729.81 -1.000000E+10 -1.000000E+10 -1.000000E+10 -1.000000E+10 -1.000000E+10  0.000000E+00  0.000000E+00  0.000000E+00  0.000000E+00  0.000000E+00  0.000000E+00  0.000000E+00  0.000000E+00  0.000000E+00  0.000000E+00  0.000000E+00  0.000000E+00       Funtag_D
      25  5.641353E+05  5.272783E+05       36857.0       19.1857     0.0507669      0.382357      0.211619      0.235929     0.0299601    0.00841385    0.00740981      0.992358       Opttag_D
      25       51.6550       2.24577       1.00000  0.000000E+00       1.00000  0.000000E+00      0.831206       52.4533       1.00000       22.4185  0.000000E+00      0.262190  0.000000E+00      0.262190  0.000000E+00  0.000000E+00     -0.524379       52.0422       1.49295        1       Scatag_D
      25       1541.40       5366.58  0.000000E+00  0.000000E+00       2134.36  0.000000E+00       2818.23       5334.79  0.000000E+00       9866.06       64.0876       9731.49 -1.000000E+10 -1.000000E+10 -1.000000E+10 -1.000000E+10 -1.000000E+10  0.000000E+00  0.000000E+00  0.000000E+00  0.000000E+00  0.000000E+00  0.000000E+00  0.000000E+00  0.000000E+00  0.000000E+00  0.000000E+00  0.000000E+00  0.000000E+00       Funtag_D
      26  5.640392E+05  5.271510E+05       36888.2       16.3041     0.0519163      0.389802      0.211515      0.235876     0.0302483    0.00849345    0.00741249      0.993135       Opttag_D
      26       51.6785       2.24443       1.00000  0.000000E+00       1.00000  0.000000E+00      0.831144       52.4944       1.00000       22.4232  0.000000E+00      0.257166  0.000000E+00      0.257166  0.000000E+00  0.000000E+00     -0.514331       52.0422       1.49295        1       Scatag_D
      26       1542.51       5374.99  0.000000E+00  0.000000E+00       2134.62  0.000000E+00       2827.00       5307.79  0.000000E+00       9899.32       65.1714       9736.76 -1.000000E+10 -1.000000E+10 -1.000000E+10 -1.000000E+10 -1.000000E+10  0.000000E+00  0.000000E+00  0.000000E+00  0.000000E+00  0.000000E+00  0.000000E+00  0.000000E+00  0.000000E+00  0.000000E+00  0.000000E+00  0.000000E+00  0.000000E+00       Funtag_D
      27  5.639461E+05  5.269573E+05       36988.8       18.2827     0.0539267      0.406845      0.211346      0.235787     0.0306866    0.00861169    0.00742204      0.995209       Opttag_D
      27       51.7198       2.24320       1.00000  0.000000E+00       1.00000  0.000000E+00      0.831043       52.5723       1.00000       22.4282  0.000000E+00      0.248284  0.000000E+00      0.248284  0.000000E+00  0.000000E+00     -0.496567       52.0422       1.49295        1       Scatag_D
      27       1546.49       5397.46  0.000000E+00  0.000000E+00       2135.34  0.000000E+00       2842.31       5294.48  0.000000E+00       9957.50       66.3302       9748.87 -1.000000E+10 -1.000000E+10 -1.000000E+10 -1.000000E+10 -1.000000E+10  0.000000E+00  0.000000E+00  0.000000E+00  0.000000E+00  0.000000E+00  0.000000E+00  0.000000E+00  0.000000E+00  0.000000E+00  0.000000E+00  0.000000E+00  0.000000E+00       Funtag_D
      28  5.638962E+05  5.265925E+05       37303.6       45.0389     0.0580682      0.443416      0.211017      0.235599     0.0315122    0.00883634    0.00748450       1.00580       Opttag_D
      28       51.8125       2.24293       1.00000  0.000000E+00       1.00000  0.000000E+00      0.830858       52.7441       1.00000       22.4349  0.000000E+00      0.227773  0.000000E+00      0.227773  0.000000E+00  0.000000E+00     -0.455547       52.0422       1.49295        1       Scatag_D
      28       1572.63       5512.97  0.000000E+00  0.000000E+00       2137.50  0.000000E+00       2880.02       5286.24  0.000000E+00       10071.1       67.8140       9775.39 -1.000000E+10 -1.000000E+10 -1.000000E+10 -1.000000E+10 -1.000000E+10  0.000000E+00  0.000000E+00  0.000000E+00  0.000000E+00  0.000000E+00  0.000000E+00  0.000000E+00  0.000000E+00  0.000000E+00  0.000000E+00  0.000000E+00  0.000000E+00       Funtag_D
      29  5.637536E+05  5.265359E+05       37217.7       17.0006     0.0587764      0.449632      0.210965      0.235569     0.0316403    0.00886371    0.00744467      0.999021       Opttag_D
      29       51.8290       2.24321       1.00000  0.000000E+00       1.00000  0.000000E+00      0.830828       52.7767       1.00000       22.4350  0.000000E+00      0.224079  0.000000E+00      0.224079  0.000000E+00  0.000000E+00     -0.448158       52.0422       1.49295        1       Scatag_D
      29       1555.93       5438.89  0.000000E+00  0.000000E+00       2137.77  0.000000E+00       2886.52       5264.28  0.000000E+00       10089.0       67.8157       9777.47 -1.000000E+10 -1.000000E+10 -1.000000E+10 -1.000000E+10 -1.000000E+10  0.000000E+00  0.000000E+00  0.000000E+00  0.000000E+00  0.000000E+00  0.000000E+00  0.000000E+00  0.000000E+00  0.000000E+00  0.000000E+00  0.000000E+00  0.000000E+00       Funtag_D
      30  5.636899E+05  5.264837E+05       37206.2       13.0186     0.0593608      0.454844      0.210915      0.235536     0.0317584    0.00890581    0.00743734      0.999006       Opttag_D
      30       51.8454       2.24372       1.00000  0.000000E+00       1.00000  0.000000E+00      0.830806       52.8079       1.00000       22.4335  0.000000E+00      0.219982  0.000000E+00      0.219982  0.000000E+00  0.000000E+00     -0.439964       52.0422       1.49295        1       Scatag_D
      30       1552.87       5438.73  0.000000E+00  0.000000E+00       2137.99  0.000000E+00       2893.06       5237.87  0.000000E+00       10100.0       67.5418       9778.17 -1.000000E+10 -1.000000E+10 -1.000000E+10 -1.000000E+10 -1.000000E+10  0.000000E+00  0.000000E+00  0.000000E+00  0.000000E+00  0.000000E+00  0.000000E+00  0.000000E+00  0.000000E+00  0.000000E+00  0.000000E+00  0.000000E+00  0.000000E+00       Funtag_D
      31  5.636085E+05  5.263446E+05       37264.0       16.0762     0.0609941      0.469036      0.210780      0.235456     0.0320733    0.00900531    0.00744060       1.00035       Opttag_D
      31       51.8885       2.24483       1.00000  0.000000E+00       1.00000  0.000000E+00      0.830746       52.8881       1.00000       22.4317  0.000000E+00      0.209797  0.000000E+00      0.209797  0.000000E+00  0.000000E+00     -0.419594       52.0422       1.49295        1       Scatag_D
      31       1554.23       5453.38  0.000000E+00  0.000000E+00       2138.87  0.000000E+00       2910.80       5220.78  0.000000E+00       10134.5       67.5368       9783.86 -1.000000E+10 -1.000000E+10 -1.000000E+10 -1.000000E+10 -1.000000E+10  0.000000E+00  0.000000E+00  0.000000E+00  0.000000E+00  0.000000E+00  0.000000E+00  0.000000E+00  0.000000E+00  0.000000E+00  0.000000E+00  0.000000E+00  0.000000E+00       Funtag_D
      32  5.635077E+05  5.261234E+05       37384.2       24.7052     0.0634791      0.490037      0.210570      0.235334     0.0325737    0.00917279    0.00746863       1.00325       Opttag_D
      32       51.9553       2.24641       1.00000  0.000000E+00       1.00000  0.000000E+00      0.830667       53.0087       1.00000       22.4285  0.000000E+00      0.194564  0.000000E+00      0.194564  0.000000E+00  0.000000E+00     -0.389128       52.0422       1.49295        1       Scatag_D
      32       1565.97       5485.06  0.000000E+00  0.000000E+00       2140.60  0.000000E+00       2936.86       5215.19  0.000000E+00       10181.1       68.1341       9791.36 -1.000000E+10 -1.000000E+10 -1.000000E+10 -1.000000E+10 -1.000000E+10  0.000000E+00  0.000000E+00  0.000000E+00  0.000000E+00  0.000000E+00  0.000000E+00  0.000000E+00  0.000000E+00  0.000000E+00  0.000000E+00  0.000000E+00  0.000000E+00       Funtag_D
      33  5.634198E+05  5.258627E+05       37557.1       20.3270     0.0669511      0.518220      0.210311      0.235169     0.0331639    0.00938535    0.00750397       1.00478       Opttag_D
      33       52.0468       2.24992       1.00000  0.000000E+00       1.00000  0.000000E+00      0.830575       53.1751       1.00000       22.4231  0.000000E+00      0.174863  0.000000E+00      0.174863  0.000000E+00  0.000000E+00     -0.349726       52.0422       1.49295        1       Scatag_D
      33       1580.82       5501.74  0.000000E+00  0.000000E+00       2143.75  0.000000E+00       2973.23       5232.67  0.000000E+00       10249.8       68.9881       9806.13 -1.000000E+10 -1.000000E+10 -1.000000E+10 -1.000000E+10 -1.000000E+10  0.000000E+00  0.000000E+00  0.000000E+00  0.000000E+00  0.000000E+00  0.000000E+00  0.000000E+00  0.000000E+00  0.000000E+00  0.000000E+00  0.000000E+00  0.000000E+00       Funtag_D
      34  5.633538E+05  5.258270E+05       37526.8       12.1055     0.0673062      0.520797      0.210275      0.235136     0.0332445    0.00945851    0.00750044       1.00390       Opttag_D
      34       52.0583       2.25038       1.00000  0.000000E+00       1.00000  0.000000E+00      0.830574       53.1947       1.00000       22.4186  0.000000E+00      0.172948  0.000000E+00      0.172948  0.000000E+00  0.000000E+00     -0.345895       52.0422       1.49295        1       Scatag_D
      34       1579.33       5492.12  0.000000E+00  0.000000E+00       2144.18  0.000000E+00       2973.70       5215.20  0.000000E+00       10250.1       68.9962       9803.18 -1.000000E+10 -1.000000E+10 -1.000000E+10 -1.000000E+10 -1.000000E+10  0.000000E+00  0.000000E+00  0.000000E+00  0.000000E+00  0.000000E+00  0.000000E+00  0.000000E+00  0.000000E+00  0.000000E+00  0.000000E+00  0.000000E+00  0.000000E+00       Funtag_D
      35  5.632867E+05  5.257719E+05       37514.8       12.8870     0.0679177      0.525002      0.210217      0.235075     0.0333694    0.00960079    0.00750529       1.00384       Opttag_D
      35       52.0789       2.25154       1.00000  0.000000E+00       1.00000  0.000000E+00      0.830577       53.2307       1.00000       22.4082  0.000000E+00      0.169820  0.000000E+00      0.169820  0.000000E+00  0.000000E+00     -0.339640       52.0422       1.49295        1       Scatag_D
      35       1581.38       5491.50  0.000000E+00  0.000000E+00       2144.91  0.000000E+00       2972.99       5206.98  0.000000E+00       10249.5       69.0089       9798.53 -1.000000E+10 -1.000000E+10 -1.000000E+10 -1.000000E+10 -1.000000E+10  0.000000E+00  0.000000E+00  0.000000E+00  0.000000E+00  0.000000E+00  0.000000E+00  0.000000E+00  0.000000E+00  0.000000E+00  0.000000E+00  0.000000E+00  0.000000E+00       Funtag_D
      36  5.632361E+05  5.256873E+05       37548.7       16.2963     0.0692176      0.534148      0.210123      0.234983     0.0335607    0.00975286    0.00751421       1.00467       Opttag_D
      36       52.1173       2.25417       1.00000  0.000000E+00       1.00000  0.000000E+00      0.830567       53.3011       1.00000       22.3941  0.000000E+00      0.163047  0.000000E+00      0.163047  0.000000E+00  0.000000E+00     -0.326094       52.0422       1.49295        1       Scatag_D
      36       1585.14       5500.52  0.000000E+00  0.000000E+00       2146.37  0.000000E+00       2977.17       5211.06  0.000000E+00       10262.1       69.0494       9797.36 -1.000000E+10 -1.000000E+10 -1.000000E+10 -1.000000E+10 -1.000000E+10  0.000000E+00  0.000000E+00  0.000000E+00  0.000000E+00  0.000000E+00  0.000000E+00  0.000000E+00  0.000000E+00  0.000000E+00  0.000000E+00  0.000000E+00  0.000000E+00       Funtag_D
      37  5.631719E+05  5.256051E+05       37566.9       12.8472     0.0707858      0.544553      0.210022      0.234877     0.0337468    0.00990998    0.00751183       1.00484       Opttag_D
      37       52.1632       2.25800       1.00000  0.000000E+00       1.00000  0.000000E+00      0.830557       53.3867       1.00000       22.3752  0.000000E+00      0.155017  0.000000E+00      0.155017  0.000000E+00  0.000000E+00     -0.310034       52.0422       1.49295        1       Scatag_D
      37       1584.13       5502.41  0.000000E+00  0.000000E+00       2148.28  0.000000E+00       2980.41       5208.60  0.000000E+00       10277.8       68.8642       9796.36 -1.000000E+10 -1.000000E+10 -1.000000E+10 -1.000000E+10 -1.000000E+10  0.000000E+00  0.000000E+00  0.000000E+00  0.000000E+00  0.000000E+00  0.000000E+00  0.000000E+00  0.000000E+00  0.000000E+00  0.000000E+00  0.000000E+00  0.000000E+00       Funtag_D
      38  5.631138E+05  5.255475E+05       37566.3       12.7035     0.0720568      0.552129      0.209950      0.234798     0.0338771     0.0100029    0.00750446       1.00522       Opttag_D
      38       52.1996       2.26135       1.00000  0.000000E+00       1.00000  0.000000E+00      0.830550       53.4530       1.00000       22.3585  0.000000E+00      0.148537  0.000000E+00      0.148537  0.000000E+00  0.000000E+00     -0.297075       52.0422       1.49295        1       Scatag_D
      38       1581.03       5506.57  0.000000E+00  0.000000E+00       2149.84  0.000000E+00       2978.93       5198.24  0.000000E+00       10288.6       68.5821       9794.52 -1.000000E+10 -1.000000E+10 -1.000000E+10 -1.000000E+10 -1.000000E+10  0.000000E+00  0.000000E+00  0.000000E+00  0.000000E+00  0.000000E+00  0.000000E+00  0.000000E+00  0.000000E+00  0.000000E+00  0.000000E+00  0.000000E+00  0.000000E+00       Funtag_D
      39  5.630591E+05  5.254843E+05       37574.8       13.5491     0.0734586      0.559485      0.209876      0.234726     0.0340202     0.0100507    0.00749485       1.00575       Opttag_D
      39       52.2386       2.26490       1.00000  0.000000E+00       1.00000  0.000000E+00      0.830539       53.5201       1.00000       22.3410  0.000000E+00      0.141452  0.000000E+00      0.141452  0.000000E+00  0.000000E+00     -0.282903       52.0422       1.49295        1       Scatag_D
      39       1576.98       5512.41  0.000000E+00  0.000000E+00       2151.53  0.000000E+00       2973.62       5201.81  0.000000E+00       10298.0       68.3282       9792.09 -1.000000E+10 -1.000000E+10 -1.000000E+10 -1.000000E+10 -1.000000E+10  0.000000E+00  0.000000E+00  0.000000E+00  0.000000E+00  0.000000E+00  0.000000E+00  0.000000E+00  0.000000E+00  0.000000E+00  0.000000E+00  0.000000E+00  0.000000E+00       Funtag_D
      40  5.630136E+05  5.254535E+05       37560.1       12.0742     0.0739814      0.561022      0.209849      0.234701     0.0340898     0.0100489    0.00749597       1.00539       Opttag_D
      40       52.2530       2.26592       1.00000  0.000000E+00       1.00000  0.000000E+00      0.830534       53.5378       1.00000       22.3340  0.000000E+00      0.139016  0.000000E+00      0.139016  0.000000E+00  0.000000E+00     -0.278032       52.0422       1.49295        1       Scatag_D
      40       1577.45       5508.49  0.000000E+00  0.000000E+00       2152.19  0.000000E+00       2966.42       5201.69  0.000000E+00       10296.8       68.2339       9788.80 -1.000000E+10 -1.000000E+10 -1.000000E+10 -1.000000E+10 -1.000000E+10  0.000000E+00  0.000000E+00  0.000000E+00  0.000000E+00  0.000000E+00  0.000000E+00  0.000000E+00  0.000000E+00  0.000000E+00  0.000000E+00  0.000000E+00  0.000000E+00       Funtag_D
      41  5.629649E+05  5.254184E+05       37546.4       10.5714     0.0745612      0.561984      0.209818      0.234677     0.0341692     0.0100267    0.00750138       1.00501       Opttag_D
      41       52.2687       2.26688       1.00000  0.000000E+00       1.00000  0.000000E+00      0.830527       53.5525       1.00000       22.3275  0.000000E+00      0.136745  0.000000E+00      0.136745  0.000000E+00  0.000000E+00     -0.273491       52.0422       1.49295        1       Scatag_D
      41       1579.73       5504.31  0.000000E+00  0.000000E+00       2152.82  0.000000E+00       2957.39       5205.73  0.000000E+00       10293.6       68.1444       9784.73 -1.000000E+10 -1.000000E+10 -1.000000E+10 -1.000000E+10 -1.000000E+10  0.000000E+00  0.000000E+00  0.000000E+00  0.000000E+00  0.000000E+00  0.000000E+00  0.000000E+00  0.000000E+00  0.000000E+00  0.000000E+00  0.000000E+00  0.000000E+00       Funtag_D
      42  5.629218E+05  5.253833E+05       37538.4       14.6039     0.0755068      0.563660      0.209778      0.234636     0.0342486     0.0100194    0.00750960       1.00492       Opttag_D
      42       52.2940       2.26903       1.00000  0.000000E+00       1.00000  0.000000E+00      0.830519       53.5801       1.00000       22.3183  0.000000E+00      0.133868  0.000000E+00      0.133868  0.000000E+00  0.000000E+00     -0.267736       52.0422       1.49295        1       Scatag_D
      42       1583.19       5503.26  0.000000E+00  0.000000E+00       2153.90  0.000000E+00       2948.02       5209.39  0.000000E+00       10291.7       67.7712       9781.21 -1.000000E+10 -1.000000E+10 -1.000000E+10 -1.000000E+10 -1.000000E+10  0.000000E+00  0.000000E+00  0.000000E+00  0.000000E+00  0.000000E+00  0.000000E+00  0.000000E+00  0.000000E+00  0.000000E+00  0.000000E+00  0.000000E+00  0.000000E+00       Funtag_D
      43  5.628819E+05  5.253652E+05       37516.7       11.7457     0.0764456      0.565041      0.209750      0.234592     0.0342896     0.0100203    0.00751063       1.00455       Opttag_D
      43       52.3188       2.27164       1.00000  0.000000E+00       1.00000  0.000000E+00      0.830513       53.6092       1.00000       22.3099  0.000000E+00      0.131366  0.000000E+00      0.131366  0.000000E+00  0.000000E+00     -0.262732       52.0422       1.49295        1       Scatag_D
      43       1583.63       5499.24  0.000000E+00  0.000000E+00       2155.06  0.000000E+00       2941.39       5198.69  0.000000E+00       10292.4       67.2805       9778.95 -1.000000E+10 -1.000000E+10 -1.000000E+10 -1.000000E+10 -1.000000E+10  0.000000E+00  0.000000E+00  0.000000E+00  0.000000E+00  0.000000E+00  0.000000E+00  0.000000E+00  0.000000E+00  0.000000E+00  0.000000E+00  0.000000E+00  0.000000E+00       Funtag_D
      44  5.628492E+05  5.253622E+05       37486.9       8.71222     0.0770153      0.566920      0.209736      0.234558     0.0342964     0.0100360    0.00749933       1.00419       Opttag_D
      44       52.3337       2.27341       1.00000  0.000000E+00       1.00000  0.000000E+00      0.830507       53.6289       1.00000       22.3064  0.000000E+00      0.130029  0.000000E+00      0.130029  0.000000E+00  0.000000E+00     -0.260059       52.0422       1.49295        1       Scatag_D
      44       1578.86       5495.31  0.000000E+00  0.000000E+00       2155.72  0.000000E+00       2939.79       5179.13  0.000000E+00       10293.9       66.8807       9777.38 -1.000000E+10 -1.000000E+10 -1.000000E+10 -1.000000E+10 -1.000000E+10  0.000000E+00  0.000000E+00  0.000000E+00  0.000000E+00  0.000000E+00  0.000000E+00  0.000000E+00  0.000000E+00  0.000000E+00  0.000000E+00  0.000000E+00  0.000000E+00       Funtag_D
      45  5.628103E+05  5.253550E+05       37455.3       9.98838     0.0780729      0.575550      0.209708      0.234499     0.0343128     0.0100686    0.00748008       1.00400       Opttag_D
      45       52.3606       2.27661       1.00000  0.000000E+00       1.00000  0.000000E+00      0.830491       53.6640       1.00000       22.3019  0.000000E+00      0.127234  0.000000E+00      0.127234  0.000000E+00  0.000000E+00     -0.254469       52.0422       1.49295        1       Scatag_D
      45       1570.77       5493.22  0.000000E+00  0.000000E+00       2156.94  0.000000E+00       2937.59       5159.07  0.000000E+00       10296.3       66.2658       9775.16 -1.000000E+10 -1.000000E+10 -1.000000E+10 -1.000000E+10 -1.000000E+10  0.000000E+00  0.000000E+00  0.000000E+00  0.000000E+00  0.000000E+00  0.000000E+00  0.000000E+00  0.000000E+00  0.000000E+00  0.000000E+00  0.000000E+00  0.000000E+00       Funtag_D
      46  5.627918E+05  5.253395E+05       37452.4       18.1507     0.0796165      0.587993      0.209673      0.234422     0.0343479     0.0100818    0.00747153       1.00406       Opttag_D
      46       52.3983       2.28086       1.00000  0.000000E+00       1.00000  0.000000E+00      0.830466       53.7082       1.00000       22.2984  0.000000E+00      0.122900  0.000000E+00      0.122900  0.000000E+00  0.000000E+00     -0.245799       52.0422       1.49295        1       Scatag_D
      46       1567.18       5493.94  0.000000E+00  0.000000E+00       2159.09  0.000000E+00       2934.60       5158.28  0.000000E+00       10299.8       65.6019       9773.89 -1.000000E+10 -1.000000E+10 -1.000000E+10 -1.000000E+10 -1.000000E+10  0.000000E+00  0.000000E+00  0.000000E+00  0.000000E+00  0.000000E+00  0.000000E+00  0.000000E+00  0.000000E+00  0.000000E+00  0.000000E+00  0.000000E+00  0.000000E+00       Funtag_D
      47  5.627587E+05  5.253294E+05       37429.3       8.85057     0.0796902      0.588625      0.209667      0.234423     0.0343706     0.0100901    0.00746205       1.00341       Opttag_D
      47       52.3998       2.28069       1.00000  0.000000E+00       1.00000  0.000000E+00      0.830460       53.7077       1.00000       22.3005  0.000000E+00      0.122402  0.000000E+00      0.122402  0.000000E+00  0.000000E+00     -0.244803       52.0422       1.49295        1       Scatag_D
      47       1563.21       5486.84  0.000000E+00  0.000000E+00       2159.08  0.000000E+00       2934.97       5148.46  0.000000E+00       10298.4       65.6607       9772.67 -1.000000E+10 -1.000000E+10 -1.000000E+10 -1.000000E+10 -1.000000E+10  0.000000E+00  0.000000E+00  0.000000E+00  0.000000E+00  0.000000E+00  0.000000E+00  0.000000E+00  0.000000E+00  0.000000E+00  0.000000E+00  0.000000E+00  0.000000E+00       Funtag_D
      48  5.627314E+05  5.253084E+05       37423.1       7.43525     0.0801334      0.592221      0.209651      0.234410     0.0344183     0.0100963    0.00745667       1.00329       Opttag_D
      48       52.4097       2.28119       1.00000  0.000000E+00       1.00000  0.000000E+00      0.830444       53.7141       1.00000       22.3037  0.000000E+00      0.120701  0.000000E+00      0.120701  0.000000E+00  0.000000E+00     -0.241401       52.0422       1.49295        1       Scatag_D
      48       1560.96       5485.46  0.000000E+00  0.000000E+00       2159.59  0.000000E+00       2934.71       5148.82  0.000000E+00       10296.9       65.6757       9770.97 -1.000000E+10 -1.000000E+10 -1.000000E+10 -1.000000E+10 -1.000000E+10  0.000000E+00  0.000000E+00  0.000000E+00  0.000000E+00  0.000000E+00  0.000000E+00  0.000000E+00  0.000000E+00  0.000000E+00  0.000000E+00  0.000000E+00  0.000000E+00       Funtag_D
      49  5.627054E+05  5.252855E+05       37419.9       8.68174     0.0809739      0.598802      0.209629      0.234380     0.0344700     0.0101054    0.00745318       1.00317       Opttag_D
      49       52.4286       2.28268       1.00000  0.000000E+00       1.00000  0.000000E+00      0.830422       53.7297       1.00000       22.3068  0.000000E+00      0.118101  0.000000E+00      0.118101  0.000000E+00  0.000000E+00     -0.236202       52.0422       1.49295        1       Scatag_D
      49       1559.49       5484.14  0.000000E+00  0.000000E+00       2160.77  0.000000E+00       2934.19       5149.81  0.000000E+00       10296.7       65.5591       9769.25 -1.000000E+10 -1.000000E+10 -1.000000E+10 -1.000000E+10 -1.000000E+10  0.000000E+00  0.000000E+00  0.000000E+00  0.000000E+00  0.000000E+00  0.000000E+00  0.000000E+00  0.000000E+00  0.000000E+00  0.000000E+00  0.000000E+00  0.000000E+00       Funtag_D
      50  5.626748E+05  5.252436E+05       37431.2       19.6731     0.0826473      0.611343      0.209590      0.234328     0.0345649     0.0100944    0.00745092       1.00405       Opttag_D
      50       52.4640       2.28534       1.00000  0.000000E+00       1.00000  0.000000E+00      0.830375       53.7558       1.00000       22.3154  0.000000E+00      0.113621  0.000000E+00      0.113621  0.000000E+00  0.000000E+00     -0.227242       52.0422       1.49295        1       Scatag_D
      50       1558.55       5493.79  0.000000E+00  0.000000E+00       2163.18  0.000000E+00       2933.88       5153.03  0.000000E+00       10297.3       65.5779       9765.87 -1.000000E+10 -1.000000E+10 -1.000000E+10 -1.000000E+10 -1.000000E+10  0.000000E+00  0.000000E+00  0.000000E+00  0.000000E+00  0.000000E+00  0.000000E+00  0.000000E+00  0.000000E+00  0.000000E+00  0.000000E+00  0.000000E+00  0.000000E+00       Funtag_D
      51  5.626352E+05  5.252296E+05       37405.5       8.94086     0.0838561      0.620148      0.209570      0.234285     0.0345964     0.0101032    0.00744500       1.00296       Opttag_D
      51       52.4889       2.28773       1.00000  0.000000E+00       1.00000  0.000000E+00      0.830348       53.7767       1.00000       22.3206  0.000000E+00      0.110798  0.000000E+00      0.110798  0.000000E+00  0.000000E+00     -0.221597       52.0422       1.49295        1       Scatag_D
      51       1556.07       5481.87  0.000000E+00  0.000000E+00       2165.23  0.000000E+00       2933.87       5137.86  0.000000E+00       10300.4       65.5107       9764.71 -1.000000E+10 -1.000000E+10 -1.000000E+10 -1.000000E+10 -1.000000E+10  0.000000E+00  0.000000E+00  0.000000E+00  0.000000E+00  0.000000E+00  0.000000E+00  0.000000E+00  0.000000E+00  0.000000E+00  0.000000E+00  0.000000E+00  0.000000E+00       Funtag_D
      52  5.626137E+05  5.252246E+05       37389.2       6.64979     0.0841587      0.622158      0.209565      0.234269     0.0346080     0.0101041    0.00744116       1.00276       Opttag_D
      52       52.4941       2.28806       1.00000  0.000000E+00       1.00000  0.000000E+00      0.830337       53.7793       1.00000       22.3230  0.000000E+00      0.110433  0.000000E+00      0.110433  0.000000E+00  0.000000E+00     -0.220866       52.0422       1.49295        1       Scatag_D
      52       1554.47       5479.63  0.000000E+00  0.000000E+00       2165.82  0.000000E+00       2933.54       5125.06  0.000000E+00       10301.5       65.5730       9763.61 -1.000000E+10 -1.000000E+10 -1.000000E+10 -1.000000E+10 -1.000000E+10  0.000000E+00  0.000000E+00  0.000000E+00  0.000000E+00  0.000000E+00  0.000000E+00  0.000000E+00  0.000000E+00  0.000000E+00  0.000000E+00  0.000000E+00  0.000000E+00       Funtag_D
      53  5.625829E+05  5.252102E+05       37372.7       8.78211     0.0849163      0.627204      0.209549      0.234234     0.0346406     0.0101044    0.00743708       1.00264       Opttag_D
      53       52.5063       2.28874       1.00000  0.000000E+00       1.00000  0.000000E+00      0.830306       53.7837       1.00000       22.3304  0.000000E+00      0.109550  0.000000E+00      0.109550  0.000000E+00  0.000000E+00     -0.219100       52.0422       1.49295        1       Scatag_D
      53       1552.76       5478.41  0.000000E+00  0.000000E+00       2167.14  0.000000E+00       2932.99       5110.09  0.000000E+00       10304.2       65.7643       9761.39 -1.000000E+10 -1.000000E+10 -1.000000E+10 -1.000000E+10 -1.000000E+10  0.000000E+00  0.000000E+00  0.000000E+00  0.000000E+00  0.000000E+00  0.000000E+00  0.000000E+00  0.000000E+00  0.000000E+00  0.000000E+00  0.000000E+00  0.000000E+00       Funtag_D
      54  5.625660E+05  5.251850E+05       37381.0       16.3103     0.0861153      0.635315      0.209528      0.234192     0.0346975     0.0100672    0.00743821       1.00263       Opttag_D
      54       52.5248       2.28967       1.00000  0.000000E+00       1.00000  0.000000E+00      0.830260       53.7887       1.00000       22.3440  0.000000E+00      0.107709  0.000000E+00      0.107709  0.000000E+00  0.000000E+00     -0.215419       52.0422       1.49295        1       Scatag_D
      54       1553.23       5478.24  0.000000E+00  0.000000E+00       2169.20  0.000000E+00       2932.75       5113.91  0.000000E+00       10308.4       65.9643       9759.31 -1.000000E+10 -1.000000E+10 -1.000000E+10 -1.000000E+10 -1.000000E+10  0.000000E+00  0.000000E+00  0.000000E+00  0.000000E+00  0.000000E+00  0.000000E+00  0.000000E+00  0.000000E+00  0.000000E+00  0.000000E+00  0.000000E+00  0.000000E+00       Funtag_D
      55  5.625388E+05  5.251768E+05       37362.0       8.58122     0.0863168      0.636718      0.209524      0.234184     0.0347162     0.0100771    0.00743646       1.00238       Opttag_D
      55       52.5270       2.28954       1.00000  0.000000E+00       1.00000  0.000000E+00      0.830249       53.7866       1.00000       22.3478  0.000000E+00      0.107537  0.000000E+00      0.107537  0.000000E+00  0.000000E+00     -0.215075       52.0422       1.49295        1       Scatag_D
      55       1552.51       5475.56  0.000000E+00  0.000000E+00       2169.49  0.000000E+00       2932.55       5098.73  0.000000E+00       10308.7       65.9860       9758.45 -1.000000E+10 -1.000000E+10 -1.000000E+10 -1.000000E+10 -1.000000E+10  0.000000E+00  0.000000E+00  0.000000E+00  0.000000E+00  0.000000E+00  0.000000E+00  0.000000E+00  0.000000E+00  0.000000E+00  0.000000E+00  0.000000E+00  0.000000E+00       Funtag_D
      56  5.625130E+05  5.251576E+05       37355.4       6.92984     0.0868975      0.640797      0.209513      0.234167     0.0347596     0.0100856    0.00743621       1.00222       Opttag_D
      56       52.5341       2.28949       1.00000  0.000000E+00       1.00000  0.000000E+00      0.830221       53.7837       1.00000       22.3573  0.000000E+00      0.106754  0.000000E+00      0.106754  0.000000E+00  0.000000E+00     -0.213508       52.0422       1.49295        1       Scatag_D
      56       1552.40       5473.73  0.000000E+00  0.000000E+00       2170.35  0.000000E+00       2932.18       5094.45  0.000000E+00       10309.7       65.9939       9756.60 -1.000000E+10 -1.000000E+10 -1.000000E+10 -1.000000E+10 -1.000000E+10  0.000000E+00  0.000000E+00  0.000000E+00  0.000000E+00  0.000000E+00  0.000000E+00  0.000000E+00  0.000000E+00  0.000000E+00  0.000000E+00  0.000000E+00  0.000000E+00       Funtag_D
      57  5.624936E+05  5.251404E+05       37353.2       7.45436     0.0876219      0.645784      0.209507      0.234151     0.0347985     0.0100957    0.00743649       1.00210       Opttag_D
      57       52.5430       2.28965       1.00000  0.000000E+00       1.00000  0.000000E+00      0.830190       53.7816       1.00000       22.3683  0.000000E+00      0.105864  0.000000E+00      0.105864  0.000000E+00  0.000000E+00     -0.211728       52.0422       1.49295        1       Scatag_D
      57       1552.51       5472.43  0.000000E+00  0.000000E+00       2171.52  0.000000E+00       2931.94       5092.91  0.000000E+00       10311.3       65.9695       9754.66 -1.000000E+10 -1.000000E+10 -1.000000E+10 -1.000000E+10 -1.000000E+10  0.000000E+00  0.000000E+00  0.000000E+00  0.000000E+00  0.000000E+00  0.000000E+00  0.000000E+00  0.000000E+00  0.000000E+00  0.000000E+00  0.000000E+00  0.000000E+00       Funtag_D
      58  5.624719E+05  5.251130E+05       37359.0       16.4351     0.0892612      0.658083      0.209498      0.234137     0.0348604     0.0101077    0.00743009       1.00251       Opttag_D
      58       52.5616       2.29014       1.00000  0.000000E+00       1.00000  0.000000E+00      0.830122       53.7763       1.00000       22.3929  0.000000E+00      0.104611  0.000000E+00      0.104611  0.000000E+00  0.000000E+00     -0.209221       52.0422       1.49295        1       Scatag_D
      58       1549.84       5477.00  0.000000E+00  0.000000E+00       2174.08  0.000000E+00       2931.51       5096.21  0.000000E+00       10314.5       65.8721       9749.96 -1.000000E+10 -1.000000E+10 -1.000000E+10 -1.000000E+10 -1.000000E+10  0.000000E+00  0.000000E+00  0.000000E+00  0.000000E+00  0.000000E+00  0.000000E+00  0.000000E+00  0.000000E+00  0.000000E+00  0.000000E+00  0.000000E+00  0.000000E+00       Funtag_D
      59  5.624426E+05  5.251057E+05       37336.9       7.25253     0.0900601      0.671260      0.209497      0.234133     0.0348769     0.0101204    0.00742439       1.00209       Opttag_D
      59       52.5702       2.29050       1.00000  0.000000E+00       1.00000  0.000000E+00      0.830091       53.7744       1.00000       22.4046  0.000000E+00      0.104202  0.000000E+00      0.104202  0.000000E+00  0.000000E+00     -0.208403       52.0422       1.49295        1       Scatag_D
      59       1547.47       5472.35  0.000000E+00  0.000000E+00       2175.45  0.000000E+00       2931.49       5079.80  0.000000E+00       10316.6       65.8677       9747.87 -1.000000E+10 -1.000000E+10 -1.000000E+10 -1.000000E+10 -1.000000E+10  0.000000E+00  0.000000E+00  0.000000E+00  0.000000E+00  0.000000E+00  0.000000E+00  0.000000E+00  0.000000E+00  0.000000E+00  0.000000E+00  0.000000E+00  0.000000E+00       Funtag_D
      60  5.624276E+05  5.251034E+05       37324.1       5.93015     0.0902397      0.674869      0.209498      0.234137     0.0348821     0.0101312    0.00741701       1.00204       Opttag_D
      60       52.5712       2.29040       1.00000  0.000000E+00       1.00000  0.000000E+00      0.830082       53.7731       1.00000       22.4070  0.000000E+00      0.104461  0.000000E+00      0.104461  0.000000E+00  0.000000E+00     -0.208921       52.0422       1.49295        1       Scatag_D
      60       1544.39       5471.84  0.000000E+00  0.000000E+00       2175.72  0.000000E+00       2931.24       5072.48  0.000000E+00       10316.4       65.8987       9746.16 -1.000000E+10 -1.000000E+10 -1.000000E+10 -1.000000E+10 -1.000000E+10  0.000000E+00  0.000000E+00  0.000000E+00  0.000000E+00  0.000000E+00  0.000000E+00  0.000000E+00  0.000000E+00  0.000000E+00  0.000000E+00  0.000000E+00  0.000000E+00       Funtag_D
      61  5.624059E+05  5.250905E+05       37315.4       7.66642     0.0908648      0.686563      0.209495      0.234147     0.0349112     0.0101308    0.00740622       1.00214       Opttag_D
      61       52.5754       2.29007       1.00000  0.000000E+00       1.00000  0.000000E+00      0.830054       53.7688       1.00000       22.4163  0.000000E+00      0.104882  0.000000E+00      0.104882  0.000000E+00  0.000000E+00     -0.209763       52.0422       1.49295        1       Scatag_D
      61       1539.90       5472.89  0.000000E+00  0.000000E+00       2176.66  0.000000E+00       2931.31       5068.89  0.000000E+00       10316.9       66.0546       9742.78 -1.000000E+10 -1.000000E+10 -1.000000E+10 -1.000000E+10 -1.000000E+10  0.000000E+00  0.000000E+00  0.000000E+00  0.000000E+00  0.000000E+00  0.000000E+00  0.000000E+00  0.000000E+00  0.000000E+00  0.000000E+00  0.000000E+00  0.000000E+00       Funtag_D
      62  5.623905E+05  5.250615E+05       37329.0       13.9876     0.0920563      0.708024      0.209483      0.234159     0.0349770     0.0101280    0.00740303       1.00252       Opttag_D
      62       52.5837       2.28935       1.00000  0.000000E+00       1.00000  0.000000E+00      0.830003       53.7598       1.00000       22.4343  0.000000E+00      0.105327  0.000000E+00      0.105327  0.000000E+00  0.000000E+00     -0.210653       52.0422       1.49295        1       Scatag_D
      62       1538.58       5477.05  0.000000E+00  0.000000E+00       2178.56  0.000000E+00       2932.88       5077.60  0.000000E+00       10319.1       66.3754       9738.87 -1.000000E+10 -1.000000E+10 -1.000000E+10 -1.000000E+10 -1.000000E+10  0.000000E+00  0.000000E+00  0.000000E+00  0.000000E+00  0.000000E+00  0.000000E+00  0.000000E+00  0.000000E+00  0.000000E+00  0.000000E+00  0.000000E+00  0.000000E+00       Funtag_D
      63  5.623682E+05  5.250439E+05       37324.2       7.24903     0.0924233      0.714663      0.209473      0.234163     0.0350167     0.0101023    0.00740442       1.00251       Opttag_D
      63       52.5858       2.28878       1.00000  0.000000E+00       1.00000  0.000000E+00      0.829985       53.7556       1.00000       22.4407  0.000000E+00      0.105311  0.000000E+00      0.105311  0.000000E+00  0.000000E+00     -0.210622       52.0422       1.49295        1       Scatag_D
      63       1539.16       5476.94  0.000000E+00  0.000000E+00       2179.14  0.000000E+00       2933.81       5071.17  0.000000E+00       10319.7       66.4807       9737.80 -1.000000E+10 -1.000000E+10 -1.000000E+10 -1.000000E+10 -1.000000E+10  0.000000E+00  0.000000E+00  0.000000E+00  0.000000E+00  0.000000E+00  0.000000E+00  0.000000E+00  0.000000E+00  0.000000E+00  0.000000E+00  0.000000E+00  0.000000E+00       Funtag_D
      64  5.623486E+05  5.250111E+05       37337.5       6.14373     0.0931058      0.726723      0.209456      0.234167     0.0350912     0.0100664    0.00741340       1.00272       Opttag_D
      64       52.5898       2.28775       1.00000  0.000000E+00       1.00000  0.000000E+00      0.829955       53.7478       1.00000       22.4526  0.000000E+00      0.105158  0.000000E+00      0.105158  0.000000E+00  0.000000E+00     -0.210316       52.0422       1.49295        1       Scatag_D
      64       1542.89       5479.26  0.000000E+00  0.000000E+00       2180.30  0.000000E+00       2935.79       5075.38  0.000000E+00       10321.1       66.6264       9736.18 -1.000000E+10 -1.000000E+10 -1.000000E+10 -1.000000E+10 -1.000000E+10  0.000000E+00  0.000000E+00  0.000000E+00  0.000000E+00  0.000000E+00  0.000000E+00  0.000000E+00  0.000000E+00  0.000000E+00  0.000000E+00  0.000000E+00  0.000000E+00       Funtag_D
      65  5.623324E+05  5.249753E+05       37357.1       6.68221     0.0939193      0.740988      0.209439      0.234173     0.0351722     0.0100385    0.00742698       1.00301       Opttag_D
      65       52.5944       2.28659       1.00000  0.000000E+00       1.00000  0.000000E+00      0.829920       53.7385       1.00000       22.4664  0.000000E+00      0.105102  0.000000E+00      0.105102  0.000000E+00  0.000000E+00     -0.210205       52.0422       1.49295        1       Scatag_D
      65       1548.55       5482.41  0.000000E+00  0.000000E+00       2181.75  0.000000E+00       2938.54       5081.97  0.000000E+00       10322.8       66.7246       9734.33 -1.000000E+10 -1.000000E+10 -1.000000E+10 -1.000000E+10 -1.000000E+10  0.000000E+00  0.000000E+00  0.000000E+00  0.000000E+00  0.000000E+00  0.000000E+00  0.000000E+00  0.000000E+00  0.000000E+00  0.000000E+00  0.000000E+00  0.000000E+00       Funtag_D
      66  5.623204E+05  5.249010E+05       37419.4       16.8308     0.0957052      0.771875      0.209405      0.234190     0.0353401    0.00998026    0.00746282       1.00426       Opttag_D
      66       52.6037       2.28410       1.00000  0.000000E+00       1.00000  0.000000E+00      0.829849       53.7181       1.00000       22.4958  0.000000E+00      0.105388  0.000000E+00      0.105388  0.000000E+00  0.000000E+00     -0.210776       52.0422       1.49295        1       Scatag_D
      66       1563.53       5496.06  0.000000E+00  0.000000E+00       2185.15  0.000000E+00       2945.95       5105.51  0.000000E+00       10326.6       66.8079       9729.72 -1.000000E+10 -1.000000E+10 -1.000000E+10 -1.000000E+10 -1.000000E+10  0.000000E+00  0.000000E+00  0.000000E+00  0.000000E+00  0.000000E+00  0.000000E+00  0.000000E+00  0.000000E+00  0.000000E+00  0.000000E+00  0.000000E+00  0.000000E+00       Funtag_D
      67  5.622940E+05  5.248858E+05       37408.2       6.32959     0.0961712      0.779841      0.209398      0.234195     0.0353746    0.00997486    0.00746984       1.00404       Opttag_D
      67       52.6060       2.28357       1.00000  0.000000E+00       1.00000  0.000000E+00      0.829831       53.7129       1.00000       22.5032  0.000000E+00      0.105652  0.000000E+00      0.105652  0.000000E+00  0.000000E+00     -0.211304       52.0422       1.49295        1       Scatag_D
      67       1566.47       5493.69  0.000000E+00  0.000000E+00       2186.14  0.000000E+00       2948.05       5091.28  0.000000E+00       10327.8       66.8194       9727.98 -1.000000E+10 -1.000000E+10 -1.000000E+10 -1.000000E+10 -1.000000E+10  0.000000E+00  0.000000E+00  0.000000E+00  0.000000E+00  0.000000E+00  0.000000E+00  0.000000E+00  0.000000E+00  0.000000E+00  0.000000E+00  0.000000E+00  0.000000E+00       Funtag_D
      68  5.622822E+05  5.248810E+05       37401.3       5.11319     0.0963141      0.782545      0.209396      0.234197     0.0353855    0.00998084    0.00747087       1.00406       Opttag_D
      68       52.6063       2.28336       1.00000  0.000000E+00       1.00000  0.000000E+00      0.829826       53.7110       1.00000       22.5046  0.000000E+00      0.106001  0.000000E+00      0.106001  0.000000E+00  0.000000E+00     -0.212002       52.0422       1.49295        1       Scatag_D
      68       1566.90       5493.92  0.000000E+00  0.000000E+00       2186.58  0.000000E+00       2949.12       5084.39  0.000000E+00       10327.6       66.7563       9726.01 -1.000000E+10 -1.000000E+10 -1.000000E+10 -1.000000E+10 -1.000000E+10  0.000000E+00  0.000000E+00  0.000000E+00  0.000000E+00  0.000000E+00  0.000000E+00  0.000000E+00  0.000000E+00  0.000000E+00  0.000000E+00  0.000000E+00  0.000000E+00       Funtag_D
      69  5.622652E+05  5.248619E+05       37403.3       6.64685     0.0969345      0.793428      0.209384      0.234211     0.0354288    0.00996370    0.00747861       1.00439       Opttag_D
      69       52.6088       2.28265       1.00000  0.000000E+00       1.00000  0.000000E+00      0.829803       53.7042       1.00000       22.5130  0.000000E+00      0.106782  0.000000E+00      0.106782  0.000000E+00  0.000000E+00     -0.213564       52.0422       1.49295        1       Scatag_D
      69       1570.15       5497.50  0.000000E+00  0.000000E+00       2188.13  0.000000E+00       2952.70       5078.94  0.000000E+00       10328.1       66.7402       9721.11 -1.000000E+10 -1.000000E+10 -1.000000E+10 -1.000000E+10 -1.000000E+10  0.000000E+00  0.000000E+00  0.000000E+00  0.000000E+00  0.000000E+00  0.000000E+00  0.000000E+00  0.000000E+00  0.000000E+00  0.000000E+00  0.000000E+00  0.000000E+00       Funtag_D
      70  5.622456E+05  5.248334E+05       37412.2       12.1849     0.0980791      0.812897      0.209369      0.234245     0.0354932    0.00989273    0.00748932       1.00509       Opttag_D
      70       52.6138       2.28163       1.00000  0.000000E+00       1.00000  0.000000E+00      0.829764       53.6922       1.00000       22.5284  0.000000E+00      0.108003  0.000000E+00      0.108003  0.000000E+00  0.000000E+00     -0.216005       52.0422       1.49295        1       Scatag_D
      70       1574.65       5505.22  0.000000E+00  0.000000E+00       2191.00  0.000000E+00       2958.59       5075.18  0.000000E+00       10329.3       66.8840       9711.39 -1.000000E+10 -1.000000E+10 -1.000000E+10 -1.000000E+10 -1.000000E+10  0.000000E+00  0.000000E+00  0.000000E+00  0.000000E+00  0.000000E+00  0.000000E+00  0.000000E+00  0.000000E+00  0.000000E+00  0.000000E+00  0.000000E+00  0.000000E+00       Funtag_D
      71  5.622235E+05  5.248064E+05       37417.1       7.23617     0.0992628      0.832307      0.209354      0.234287     0.0355544    0.00980151    0.00750025       1.00564       Opttag_D
      71       52.6193       2.28070       1.00000  0.000000E+00       1.00000  0.000000E+00      0.829723       53.6809       1.00000       22.5456  0.000000E+00      0.108854  0.000000E+00      0.108854  0.000000E+00  0.000000E+00     -0.217708       52.0422       1.49295        1       Scatag_D
      71       1579.25       5511.24  0.000000E+00  0.000000E+00       2193.84  0.000000E+00       2964.40       5066.97  0.000000E+00       10331.5       67.0550       9702.82 -1.000000E+10 -1.000000E+10 -1.000000E+10 -1.000000E+10 -1.000000E+10  0.000000E+00  0.000000E+00  0.000000E+00  0.000000E+00  0.000000E+00  0.000000E+00  0.000000E+00  0.000000E+00  0.000000E+00  0.000000E+00  0.000000E+00  0.000000E+00       Funtag_D
      72  5.622084E+05  5.247945E+05       37413.9       5.12289     0.0998086      0.840957      0.209348      0.234310     0.0355811    0.00973043    0.00750081       1.00585       Opttag_D
      72       52.6221       2.28032       1.00000  0.000000E+00       1.00000  0.000000E+00      0.829705       53.6754       1.00000       22.5538  0.000000E+00      0.108925  0.000000E+00      0.108925  0.000000E+00  0.000000E+00     -0.217850       52.0422       1.49295        1       Scatag_D
      72       1579.49       5513.55  0.000000E+00  0.000000E+00       2195.15  0.000000E+00       2965.78       5063.46  0.000000E+00       10332.1       67.1592       9697.21 -1.000000E+10 -1.000000E+10 -1.000000E+10 -1.000000E+10 -1.000000E+10  0.000000E+00  0.000000E+00  0.000000E+00  0.000000E+00  0.000000E+00  0.000000E+00  0.000000E+00  0.000000E+00  0.000000E+00  0.000000E+00  0.000000E+00  0.000000E+00       Funtag_D
      73  5.621915E+05  5.247768E+05       37414.7       7.07405      0.100689      0.854525      0.209334      0.234354     0.0356212    0.00959872    0.00750330       1.00622       Opttag_D
      73       52.6270       2.27981       1.00000  0.000000E+00       1.00000  0.000000E+00      0.829676       53.6660       1.00000       22.5670  0.000000E+00      0.108732  0.000000E+00      0.108732  0.000000E+00  0.000000E+00     -0.217464       52.0422       1.49295        1       Scatag_D
      73       1580.54       5517.57  0.000000E+00  0.000000E+00       2197.21  0.000000E+00       2966.54       5065.39  0.000000E+00       10333.0       67.2716       9687.20 -1.000000E+10 -1.000000E+10 -1.000000E+10 -1.000000E+10 -1.000000E+10  0.000000E+00  0.000000E+00  0.000000E+00  0.000000E+00  0.000000E+00  0.000000E+00  0.000000E+00  0.000000E+00  0.000000E+00  0.000000E+00  0.000000E+00  0.000000E+00       Funtag_D
      74  5.621798E+05  5.247642E+05       37415.6       8.52971      0.101539      0.867191      0.209328      0.234397     0.0356498    0.00945230    0.00750835       1.00654       Opttag_D
      74       52.6319       2.27945       1.00000  0.000000E+00       1.00000  0.000000E+00      0.829652       53.6563       1.00000       22.5797  0.000000E+00      0.108600  0.000000E+00      0.108600  0.000000E+00  0.000000E+00     -0.217201       52.0422       1.49295        1       Scatag_D
      74       1582.66       5521.11  0.000000E+00  0.000000E+00       2199.11  0.000000E+00       2966.20       5067.96  0.000000E+00       10333.9       67.2576       9677.40 -1.000000E+10 -1.000000E+10 -1.000000E+10 -1.000000E+10 -1.000000E+10  0.000000E+00  0.000000E+00  0.000000E+00  0.000000E+00  0.000000E+00  0.000000E+00  0.000000E+00  0.000000E+00  0.000000E+00  0.000000E+00  0.000000E+00  0.000000E+00       Funtag_D
      75  5.621671E+05  5.247642E+05       37402.9       5.98881      0.101694      0.869243      0.209329      0.234408     0.0356497    0.00940813    0.00750704       1.00644       Opttag_D
      75       52.6331       2.27951       1.00000  0.000000E+00       1.00000  0.000000E+00      0.829649       53.6541       1.00000       22.5819  0.000000E+00      0.108468  0.000000E+00      0.108468  0.000000E+00  0.000000E+00     -0.216935       52.0422       1.49295        1       Scatag_D
      75       1582.11       5519.98  0.000000E+00  0.000000E+00       2199.42  0.000000E+00       2964.25       5063.14  0.000000E+00       10333.0       67.1392       9673.83 -1.000000E+10 -1.000000E+10 -1.000000E+10 -1.000000E+10 -1.000000E+10  0.000000E+00  0.000000E+00  0.000000E+00  0.000000E+00  0.000000E+00  0.000000E+00  0.000000E+00  0.000000E+00  0.000000E+00  0.000000E+00  0.000000E+00  0.000000E+00       Funtag_D
      76  5.621452E+05  5.247665E+05       37378.7       5.99268      0.102476      0.879909      0.209330      0.234456     0.0356446    0.00924146    0.00750733       1.00640       Opttag_D
      76       52.6384       2.27976       1.00000  0.000000E+00       1.00000  0.000000E+00      0.829631       53.6451       1.00000       22.5930  0.000000E+00      0.108567  0.000000E+00      0.108567  0.000000E+00  0.000000E+00     -0.217133       52.0422       1.49295        1       Scatag_D
      76       1582.24       5519.52  0.000000E+00  0.000000E+00       2201.01  0.000000E+00       2959.91       5055.57  0.000000E+00       10332.1       66.8265       9661.56 -1.000000E+10 -1.000000E+10 -1.000000E+10 -1.000000E+10 -1.000000E+10  0.000000E+00  0.000000E+00  0.000000E+00  0.000000E+00  0.000000E+00  0.000000E+00  0.000000E+00  0.000000E+00  0.000000E+00  0.000000E+00  0.000000E+00  0.000000E+00       Funtag_D
      77  5.621312E+05  5.247744E+05       37356.7       7.80341      0.103341      0.898102      0.209335      0.234510     0.0356267    0.00907168    0.00750570       1.00644       Opttag_D
      77       52.6442       2.28021       1.00000  0.000000E+00       1.00000  0.000000E+00      0.829612       53.6375       1.00000       22.6052  0.000000E+00      0.108938  0.000000E+00      0.108938  0.000000E+00  0.000000E+00     -0.217876       52.0422       1.49295        1       Scatag_D
      77       1581.55       5519.94  0.000000E+00  0.000000E+00       2202.77  0.000000E+00       2956.65       5047.68  0.000000E+00       10332.2       66.6489       9649.33 -1.000000E+10 -1.000000E+10 -1.000000E+10 -1.000000E+10 -1.000000E+10  0.000000E+00  0.000000E+00  0.000000E+00  0.000000E+00  0.000000E+00  0.000000E+00  0.000000E+00  0.000000E+00  0.000000E+00  0.000000E+00  0.000000E+00  0.000000E+00       Funtag_D
      78  5.621125E+05  5.247918E+05       37320.7       6.15037      0.104498      0.928678      0.209350      0.234582     0.0355875    0.00884335    0.00750084       1.00638       Opttag_D
      78       52.6518       2.28103       1.00000  0.000000E+00       1.00000  0.000000E+00      0.829588       53.6283       1.00000       22.6218  0.000000E+00      0.109612  0.000000E+00      0.109612  0.000000E+00  0.000000E+00     -0.219224       52.0422       1.49295        1       Scatag_D
      78       1579.50       5519.31  0.000000E+00  0.000000E+00       2205.06  0.000000E+00       2952.71       5031.60  0.000000E+00       10332.8       66.4753       9633.29 -1.000000E+10 -1.000000E+10 -1.000000E+10 -1.000000E+10 -1.000000E+10  0.000000E+00  0.000000E+00  0.000000E+00  0.000000E+00  0.000000E+00  0.000000E+00  0.000000E+00  0.000000E+00  0.000000E+00  0.000000E+00  0.000000E+00  0.000000E+00       Funtag_D
      79  5.620957E+05  5.248192E+05       37276.5       9.10789      0.105870      0.964434      0.209367      0.234670     0.0355252    0.00858811    0.00749169       1.00626       Opttag_D
      79       52.6612       2.28228       1.00000  0.000000E+00       1.00000  0.000000E+00      0.829561       53.6201       1.00000       22.6425  0.000000E+00      0.110355  0.000000E+00      0.110355  0.000000E+00  0.000000E+00     -0.220710       52.0422       1.49295        1       Scatag_D
      79       1575.65       5518.05  0.000000E+00  0.000000E+00       2207.71  0.000000E+00       2947.54       5013.72  0.000000E+00       10333.5       66.3785       9613.95 -1.000000E+10 -1.000000E+10 -1.000000E+10 -1.000000E+10 -1.000000E+10  0.000000E+00  0.000000E+00  0.000000E+00  0.000000E+00  0.000000E+00  0.000000E+00  0.000000E+00  0.000000E+00  0.000000E+00  0.000000E+00  0.000000E+00  0.000000E+00       Funtag_D
      80  5.620788E+05  5.248348E+05       37244.0       5.46613      0.106521      0.981568      0.209381      0.234710     0.0354900    0.00847665    0.00748325       1.00605       Opttag_D
      80       52.6660       2.28298       1.00000  0.000000E+00       1.00000  0.000000E+00      0.829547       53.6181       1.00000       22.6528  0.000000E+00      0.110508  0.000000E+00      0.110508  0.000000E+00  0.000000E+00     -0.221016       52.0422       1.49295        1       Scatag_D
      80       1572.10       5515.73  0.000000E+00  0.000000E+00       2209.02  0.000000E+00       2944.32       4999.25  0.000000E+00       10333.3       66.4032       9603.95 -1.000000E+10 -1.000000E+10 -1.000000E+10 -1.000000E+10 -1.000000E+10  0.000000E+00  0.000000E+00  0.000000E+00  0.000000E+00  0.000000E+00  0.000000E+00  0.000000E+00  0.000000E+00  0.000000E+00  0.000000E+00  0.000000E+00  0.000000E+00       Funtag_D
      81  5.620663E+05  5.248388E+05       37227.5       5.31215      0.106716      0.987564      0.209385      0.234724     0.0354810    0.00845020    0.00747793       1.00586       Opttag_D
      81       52.6678       2.28322       1.00000  0.000000E+00       1.00000  0.000000E+00      0.829540       53.6195       1.00000       22.6567  0.000000E+00      0.110196  0.000000E+00      0.110196  0.000000E+00  0.000000E+00     -0.220392       52.0422       1.49295        1       Scatag_D
      81       1569.87       5513.57  0.000000E+00  0.000000E+00       2209.53  0.000000E+00       2941.97       4994.43  0.000000E+00       10332.3       66.4755       9599.34 -1.000000E+10 -1.000000E+10 -1.000000E+10 -1.000000E+10 -1.000000E+10  0.000000E+00  0.000000E+00  0.000000E+00  0.000000E+00  0.000000E+00  0.000000E+00  0.000000E+00  0.000000E+00  0.000000E+00  0.000000E+00  0.000000E+00  0.000000E+00       Funtag_D
      82  5.620481E+05  5.248514E+05       37196.8       9.03922      0.107553       1.01199      0.209397      0.234780     0.0354525    0.00834392    0.00747300       1.00558       Opttag_D
      82       52.6745       2.28400       1.00000  0.000000E+00       1.00000  0.000000E+00      0.829520       53.6211       1.00000       22.6718  0.000000E+00      0.109305  0.000000E+00      0.109305  0.000000E+00  0.000000E+00     -0.218611       52.0422       1.49295        1       Scatag_D
      82       1567.80       5510.58  0.000000E+00  0.000000E+00       2211.38  0.000000E+00       2935.38       4988.90  0.000000E+00       10330.7       66.5754       9585.45 -1.000000E+10 -1.000000E+10 -1.000000E+10 -1.000000E+10 -1.000000E+10  0.000000E+00  0.000000E+00  0.000000E+00  0.000000E+00  0.000000E+00  0.000000E+00  0.000000E+00  0.000000E+00  0.000000E+00  0.000000E+00  0.000000E+00  0.000000E+00       Funtag_D
      83  5.620321E+05  5.248647E+05       37167.4       7.82093      0.108580       1.04080      0.209409      0.234851     0.0354224    0.00817080    0.00747039       1.00541       Opttag_D
      83       52.6820       2.28480       1.00000  0.000000E+00       1.00000  0.000000E+00      0.829499       53.6208       1.00000       22.6897  0.000000E+00      0.108712  0.000000E+00      0.108712  0.000000E+00  0.000000E+00     -0.217424       52.0422       1.49295        1       Scatag_D
      83       1566.71       5508.65  0.000000E+00  0.000000E+00       2213.36  0.000000E+00       2929.52       4980.83  0.000000E+00       10330.0       66.5665       9571.77 -1.000000E+10 -1.000000E+10 -1.000000E+10 -1.000000E+10 -1.000000E+10  0.000000E+00  0.000000E+00  0.000000E+00  0.000000E+00  0.000000E+00  0.000000E+00  0.000000E+00  0.000000E+00  0.000000E+00  0.000000E+00  0.000000E+00  0.000000E+00       Funtag_D
      84  5.620201E+05  5.248630E+05       37157.0       5.41664      0.108791       1.04755      0.209410      0.234863     0.0354261    0.00814009    0.00746940       1.00527       Opttag_D
      84       52.6834       2.28482       1.00000  0.000000E+00       1.00000  0.000000E+00      0.829494       53.6208       1.00000       22.6935  0.000000E+00      0.108435  0.000000E+00      0.108435  0.000000E+00  0.000000E+00     -0.216870       52.0422       1.49295        1       Scatag_D
      84       1566.29       5507.14  0.000000E+00  0.000000E+00       2213.76  0.000000E+00       2928.39       4976.65  0.000000E+00       10329.7       66.5043       9568.56 -1.000000E+10 -1.000000E+10 -1.000000E+10 -1.000000E+10 -1.000000E+10  0.000000E+00  0.000000E+00  0.000000E+00  0.000000E+00  0.000000E+00  0.000000E+00  0.000000E+00  0.000000E+00  0.000000E+00  0.000000E+00  0.000000E+00  0.000000E+00       Funtag_D
      85  5.620035E+05  5.248644E+05       37139.1       5.16769      0.109529       1.06961      0.209413      0.234904     0.0354231    0.00804577    0.00746574       1.00516       Opttag_D
      85       52.6884       2.28511       1.00000  0.000000E+00       1.00000  0.000000E+00      0.829478       53.6211       1.00000       22.7064  0.000000E+00      0.107853  0.000000E+00      0.107853  0.000000E+00  0.000000E+00     -0.215706       52.0422       1.49295        1       Scatag_D
      85       1564.75       5505.90  0.000000E+00  0.000000E+00       2215.05  0.000000E+00       2925.64       4972.86  0.000000E+00       10329.3       66.3564       9559.29 -1.000000E+10 -1.000000E+10 -1.000000E+10 -1.000000E+10 -1.000000E+10  0.000000E+00  0.000000E+00  0.000000E+00  0.000000E+00  0.000000E+00  0.000000E+00  0.000000E+00  0.000000E+00  0.000000E+00  0.000000E+00  0.000000E+00  0.000000E+00       Funtag_D
      86  5.619926E+05  5.248718E+05       37120.8       7.41547      0.110399       1.09456      0.209422      0.234952     0.0354064    0.00792971    0.00745985       1.00505       Opttag_D
      86       52.6945       2.28570       1.00000  0.000000E+00       1.00000  0.000000E+00      0.829458       53.6236       1.00000       22.7216  0.000000E+00      0.107175  0.000000E+00      0.107175  0.000000E+00  0.000000E+00     -0.214349       52.0422       1.49295        1       Scatag_D
      86       1562.29       5504.75  0.000000E+00  0.000000E+00       2216.59  0.000000E+00       2923.07       4969.64  0.000000E+00       10328.6       66.2951       9549.62 -1.000000E+10 -1.000000E+10 -1.000000E+10 -1.000000E+10 -1.000000E+10  0.000000E+00  0.000000E+00  0.000000E+00  0.000000E+00  0.000000E+00  0.000000E+00  0.000000E+00  0.000000E+00  0.000000E+00  0.000000E+00  0.000000E+00  0.000000E+00       Funtag_D
      87  5.619800E+05  5.248751E+05       37105.0       5.28670      0.111030       1.11229      0.209430      0.234987     0.0353990    0.00786507    0.00745238       1.00484       Opttag_D
      87       52.6989       2.28602       1.00000  0.000000E+00       1.00000  0.000000E+00      0.829444       53.6254       1.00000       22.7331  0.000000E+00      0.106690  0.000000E+00      0.106690  0.000000E+00  0.000000E+00     -0.213381       52.0422       1.49295        1       Scatag_D
      87       1559.16       5502.43  0.000000E+00  0.000000E+00       2217.61  0.000000E+00       2922.12       4966.25  0.000000E+00       10328.0       66.2670       9543.14 -1.000000E+10 -1.000000E+10 -1.000000E+10 -1.000000E+10 -1.000000E+10  0.000000E+00  0.000000E+00  0.000000E+00  0.000000E+00  0.000000E+00  0.000000E+00  0.000000E+00  0.000000E+00  0.000000E+00  0.000000E+00  0.000000E+00  0.000000E+00       Funtag_D
      88  5.619657E+05  5.248729E+05       37092.9       5.60563      0.111857       1.13497      0.209434      0.235027     0.0354040    0.00781135    0.00744300       1.00459       Opttag_D
      88       52.7043       2.28622       1.00000  0.000000E+00       1.00000  0.000000E+00      0.829423       53.6292       1.00000       22.7487  0.000000E+00      0.105850  0.000000E+00      0.105850  0.000000E+00  0.000000E+00     -0.211700       52.0422       1.49295        1       Scatag_D
      88       1555.24       5499.64  0.000000E+00  0.000000E+00       2218.89  0.000000E+00       2922.60       4968.37  0.000000E+00       10326.5       66.2658       9535.37 -1.000000E+10 -1.000000E+10 -1.000000E+10 -1.000000E+10 -1.000000E+10  0.000000E+00  0.000000E+00  0.000000E+00  0.000000E+00  0.000000E+00  0.000000E+00  0.000000E+00  0.000000E+00  0.000000E+00  0.000000E+00  0.000000E+00  0.000000E+00       Funtag_D
      89  5.619563E+05  5.248636E+05       37092.7       6.73321      0.112471       1.15146      0.209434      0.235053     0.0354248    0.00778432    0.00744189       1.00446       Opttag_D
      89       52.7077       2.28604       1.00000  0.000000E+00       1.00000  0.000000E+00      0.829408       53.6309       1.00000       22.7609  0.000000E+00      0.105192  0.000000E+00      0.105192  0.000000E+00  0.000000E+00     -0.210384       52.0422       1.49295        1       Scatag_D
      89       1554.77       5498.30  0.000000E+00  0.000000E+00       2219.82  0.000000E+00       2923.78       4974.26  0.000000E+00       10325.0       66.2834       9530.50 -1.000000E+10 -1.000000E+10 -1.000000E+10 -1.000000E+10 -1.000000E+10  0.000000E+00  0.000000E+00  0.000000E+00  0.000000E+00  0.000000E+00  0.000000E+00  0.000000E+00  0.000000E+00  0.000000E+00  0.000000E+00  0.000000E+00  0.000000E+00       Funtag_D
      90  5.619470E+05  5.248543E+05       37092.6       5.05687      0.112668       1.15663      0.209429      0.235058     0.0354458    0.00779237    0.00744376       1.00428       Opttag_D
      90       52.7083       2.28572       1.00000  0.000000E+00       1.00000  0.000000E+00      0.829403       53.6316       1.00000       22.7648  0.000000E+00      0.104895  0.000000E+00      0.104895  0.000000E+00  0.000000E+00     -0.209791       52.0422       1.49295        1       Scatag_D
      90       1555.55       5496.35  0.000000E+00  0.000000E+00       2220.09  0.000000E+00       2925.14       4976.31  0.000000E+00       10324.0       66.3053       9528.81 -1.000000E+10 -1.000000E+10 -1.000000E+10 -1.000000E+10 -1.000000E+10  0.000000E+00  0.000000E+00  0.000000E+00  0.000000E+00  0.000000E+00  0.000000E+00  0.000000E+00  0.000000E+00  0.000000E+00  0.000000E+00  0.000000E+00  0.000000E+00       Funtag_D
      91  5.619302E+05  5.248242E+05       37106.0       5.45070      0.113251       1.17201      0.209416      0.235066     0.0355140    0.00782205    0.00745359       1.00409       Opttag_D
      91       52.7091       2.28462       1.00000  0.000000E+00       1.00000  0.000000E+00      0.829390       53.6326       1.00000       22.7758  0.000000E+00      0.104251  0.000000E+00      0.104251  0.000000E+00  0.000000E+00     -0.208501       52.0422       1.49295        1       Scatag_D
      91       1559.67       5494.28  0.000000E+00  0.000000E+00       2220.83  0.000000E+00       2929.86       4988.56  0.000000E+00       10321.5       66.3901       9524.90 -1.000000E+10 -1.000000E+10 -1.000000E+10 -1.000000E+10 -1.000000E+10  0.000000E+00  0.000000E+00  0.000000E+00  0.000000E+00  0.000000E+00  0.000000E+00  0.000000E+00  0.000000E+00  0.000000E+00  0.000000E+00  0.000000E+00  0.000000E+00       Funtag_D
      92  5.619215E+05  5.248099E+05       37111.6       7.18093      0.113767       1.18579      0.209411      0.235079     0.0355464    0.00779916    0.00745898       1.00409       Opttag_D
      92       52.7101       2.28409       1.00000  0.000000E+00       1.00000  0.000000E+00      0.829380       53.6334       1.00000       22.7844  0.000000E+00      0.104198  0.000000E+00      0.104198  0.000000E+00  0.000000E+00     -0.208396       52.0422       1.49295        1       Scatag_D
      92       1561.92       5494.22  0.000000E+00  0.000000E+00       2221.41  0.000000E+00       2932.56       4992.59  0.000000E+00       10320.6       66.4508       9521.86 -1.000000E+10 -1.000000E+10 -1.000000E+10 -1.000000E+10 -1.000000E+10  0.000000E+00  0.000000E+00  0.000000E+00  0.000000E+00  0.000000E+00  0.000000E+00  0.000000E+00  0.000000E+00  0.000000E+00  0.000000E+00  0.000000E+00  0.000000E+00       Funtag_D
      93  5.619125E+05  5.248029E+05       37109.7       5.31198      0.114026       1.19266      0.209408      0.235082     0.0355624    0.00778581    0.00745905       1.00406       Opttag_D
      93       52.7103       2.28380       1.00000  0.000000E+00       1.00000  0.000000E+00      0.829376       53.6333       1.00000       22.7883  0.000000E+00      0.104292  0.000000E+00      0.104292  0.000000E+00  0.000000E+00     -0.208584       52.0422       1.49295        1       Scatag_D
      93       1561.95       5493.91  0.000000E+00  0.000000E+00       2221.67  0.000000E+00       2933.60       4991.65  0.000000E+00       10320.3       66.4909       9520.05 -1.000000E+10 -1.000000E+10 -1.000000E+10 -1.000000E+10 -1.000000E+10  0.000000E+00  0.000000E+00  0.000000E+00  0.000000E+00  0.000000E+00  0.000000E+00  0.000000E+00  0.000000E+00  0.000000E+00  0.000000E+00  0.000000E+00  0.000000E+00       Funtag_D
      94  5.618977E+05  5.247843E+05       37113.4       4.85369      0.114841       1.21331      0.209404      0.235097     0.0356043    0.00772807    0.00746110       1.00432       Opttag_D
      94       52.7112       2.28306       1.00000  0.000000E+00       1.00000  0.000000E+00      0.829365       53.6333       1.00000       22.8000  0.000000E+00      0.104577  0.000000E+00      0.104577  0.000000E+00  0.000000E+00     -0.209154       52.0422       1.49295        1       Scatag_D
      94       1562.81       5496.76  0.000000E+00  0.000000E+00       2222.41  0.000000E+00       2936.08       4993.93  0.000000E+00       10319.9       66.6585       9514.89 -1.000000E+10 -1.000000E+10 -1.000000E+10 -1.000000E+10 -1.000000E+10  0.000000E+00  0.000000E+00  0.000000E+00  0.000000E+00  0.000000E+00  0.000000E+00  0.000000E+00  0.000000E+00  0.000000E+00  0.000000E+00  0.000000E+00  0.000000E+00       Funtag_D
      95  5.618903E+05  5.247743E+05       37115.9       5.93564      0.115284       1.22281      0.209401      0.235108     0.0356268    0.00769000    0.00746325       1.00448       Opttag_D
      95       52.7119       2.28270       1.00000  0.000000E+00       1.00000  0.000000E+00      0.829358       53.6339       1.00000       22.8063  0.000000E+00      0.104542  0.000000E+00      0.104542  0.000000E+00  0.000000E+00     -0.209085       52.0422       1.49295        1       Scatag_D
      95       1563.71       5498.49  0.000000E+00  0.000000E+00       2222.76  0.000000E+00       2936.78       4996.25  0.000000E+00       10319.5       66.7853       9511.71 -1.000000E+10 -1.000000E+10 -1.000000E+10 -1.000000E+10 -1.000000E+10  0.000000E+00  0.000000E+00  0.000000E+00  0.000000E+00  0.000000E+00  0.000000E+00  0.000000E+00  0.000000E+00  0.000000E+00  0.000000E+00  0.000000E+00  0.000000E+00       Funtag_D
      96  5.618817E+05  5.247675E+05       37114.1       5.19273      0.115642       1.22941      0.209398      0.235119     0.0356423    0.00765262    0.00746421       1.00460       Opttag_D
      96       52.7126       2.28247       1.00000  0.000000E+00       1.00000  0.000000E+00      0.829352       53.6349       1.00000       22.8108  0.000000E+00      0.104451  0.000000E+00      0.104451  0.000000E+00  0.000000E+00     -0.208902       52.0422       1.49295        1       Scatag_D
      96       1564.11       5499.80  0.000000E+00  0.000000E+00       2222.97  0.000000E+00       2936.81       4995.98  0.000000E+00       10319.0       66.9007       9508.55 -1.000000E+10 -1.000000E+10 -1.000000E+10 -1.000000E+10 -1.000000E+10  0.000000E+00  0.000000E+00  0.000000E+00  0.000000E+00  0.000000E+00  0.000000E+00  0.000000E+00  0.000000E+00  0.000000E+00  0.000000E+00  0.000000E+00  0.000000E+00       Funtag_D
      97  5.618681E+05  5.247495E+05       37118.6       8.81570      0.116841       1.24757      0.209392      0.235166     0.0356831    0.00750377    0.00747104       1.00518       Opttag_D
      97       52.7141       2.28182       1.00000  0.000000E+00       1.00000  0.000000E+00      0.829336       53.6389       1.00000       22.8254  0.000000E+00      0.104171  0.000000E+00      0.104171  0.000000E+00  0.000000E+00     -0.208343       52.0422       1.49295        1       Scatag_D
      97       1566.97       5506.13  0.000000E+00  0.000000E+00       2223.47  0.000000E+00       2936.19       5003.36  0.000000E+00       10317.8       67.2206       9497.47 -1.000000E+10 -1.000000E+10 -1.000000E+10 -1.000000E+10 -1.000000E+10  0.000000E+00  0.000000E+00  0.000000E+00  0.000000E+00  0.000000E+00  0.000000E+00  0.000000E+00  0.000000E+00  0.000000E+00  0.000000E+00  0.000000E+00  0.000000E+00       Funtag_D
      98  5.618571E+05  5.247478E+05       37109.4       5.75210      0.117270       1.25761      0.209394      0.235187     0.0356870    0.00744028    0.00747297       1.00507       Opttag_D
      98       52.7141       2.28167       1.00000  0.000000E+00       1.00000  0.000000E+00      0.829331       53.6406       1.00000       22.8300  0.000000E+00      0.104278  0.000000E+00      0.104278  0.000000E+00  0.000000E+00     -0.208556       52.0422       1.49295        1       Scatag_D
      98       1567.79       5505.00  0.000000E+00  0.000000E+00       2223.52  0.000000E+00       2935.59       4999.82  0.000000E+00       10317.7       67.2720       9492.68 -1.000000E+10 -1.000000E+10 -1.000000E+10 -1.000000E+10 -1.000000E+10  0.000000E+00  0.000000E+00  0.000000E+00  0.000000E+00  0.000000E+00  0.000000E+00  0.000000E+00  0.000000E+00  0.000000E+00  0.000000E+00  0.000000E+00  0.000000E+00       Funtag_D
      99  5.618503E+05  5.247499E+05       37100.4       4.54236      0.117238       1.25744      0.209395      0.235192     0.0356822    0.00743016    0.00747001       1.00492       Opttag_D
      99       52.7136       2.28169       1.00000  0.000000E+00       1.00000  0.000000E+00      0.829331       53.6409       1.00000       22.8290  0.000000E+00      0.104310  0.000000E+00      0.104310  0.000000E+00  0.000000E+00     -0.208620       52.0422       1.49295        1       Scatag_D
      99       1566.55       5503.32  0.000000E+00  0.000000E+00       2223.35  0.000000E+00       2934.72       4996.82  0.000000E+00       10317.6       67.2081       9490.89 -1.000000E+10 -1.000000E+10 -1.000000E+10 -1.000000E+10 -1.000000E+10  0.000000E+00  0.000000E+00  0.000000E+00  0.000000E+00  0.000000E+00  0.000000E+00  0.000000E+00  0.000000E+00  0.000000E+00  0.000000E+00  0.000000E+00  0.000000E+00       Funtag_D

   MaxLik Scaling Cycle 0105

   Wilson plot

   Statistics in resolution bins after refinement cycle 0003
```


|  |  |
| --- | --- |
|  | - Rxpct factors plot- Rxpct factors table- <Log-Lik Gain>- Ln Sigma A- Correlation Coefficients |


```
   Iters     Tot_Funct    Xray_Funct    Geom_Funct          Grms     rmsD2init     maxD2init             R         Rfree           LLG       LLGfree     rms(bond)    rms(angle)
   Iters             K   B_IMPF_FRAG        K_MISS   B_TEMP_MISS   K_IMPF_MISS   B_IMPF_MISS        K_SOLV   B_TEMP_SOLV   K_IMPF_SOLV   B_IMPF_SOLV        B_TEMP     B_TEMP_11     B_TEMP_12     B_TEMP_22     B_TEMP_13     B_TEMP_23     B_TEMP_33      K_WILSON      B_WILSON      FGCYCLES
   Iters          BOND         ANGLE      IMPROPER       TORSION    SINTORSION      TRIGONAL        PLANAR  BCORRELATION       CONTACT     IDEALDIST        CHIRAL    SIMILARITY       OCCSSUM  UTILDISTANCE     UTILANGLE   UTILTORSION        RHOFIT        ADP-01        ADP-02        ADP-03        ADP-04        ADP-05        ADP-06        ADP-07        ADP-08        ADP-09        ADP-10      RHOFITPC            QM
     100  5.618322E+05  5.247551E+05       37077.1       4.70176      0.117630       1.27981      0.209399      0.235235     0.0356704    0.00731686    0.00746406       1.00464       Opttag_D
     100       52.7116       2.28160       1.00000  0.000000E+00       1.00000  0.000000E+00      0.829325       53.6433       1.00000       22.8310  0.000000E+00      0.104513  0.000000E+00      0.104513  0.000000E+00  0.000000E+00     -0.209025       52.0422       1.49295        1       Scatag_D
     100       1564.05       5500.29  0.000000E+00  0.000000E+00       2222.73  0.000000E+00       2931.16       4994.80  0.000000E+00       10317.5       67.0548       9479.48 -1.000000E+10 -1.000000E+10 -1.000000E+10 -1.000000E+10 -1.000000E+10  0.000000E+00  0.000000E+00  0.000000E+00  0.000000E+00  0.000000E+00  0.000000E+00  0.000000E+00  0.000000E+00  0.000000E+00  0.000000E+00  0.000000E+00  0.000000E+00       Funtag_D
   Geometry Restraint Deviations SCREEN Information

 
 No -bcorrel_zeroocchydrogens_factor flag found - keeping default: will set B for OCC=0 hydrogens to    1.200 times heavy-atom B


 TLS information. Total number of groups:     2


 Group   Natoms    FixRB   FixTLS   Group Tag    Group Spec
     1     2770      Y        Y     tls1    { A|* }
     2     2767      Y        Y     tls2    { B|* }
 
NOTE BUSTER_TLS_SET tls1 { A|* }
NOTE BUSTER_TLS_T   tls1     -1.79412E-01 -8.22794E-02  2.45470E-01 -1.90737E-02  4.55243E-02  1.70868E-02
NOTE BUSTER_TLS_L   tls1      1.20658E+00  7.85380E-01  6.16119E-01  1.76434E-01 -3.54449E-01  2.45657E-01
NOTE BUSTER_TLS_S   tls1      1.76213E-01 -1.34977E-01  2.10827E-01 -7.74912E-02 -1.15771E-01 -8.80908E-02 -2.18500E-02 -3.23633E-02
NOTE BUSTER_TLS_O   tls1     -2.33295E+01 -8.34950E+01  1.14606E+01
NOTE BUSTER_TLS_FIX tls1 ALL
 
NOTE BUSTER_TLS_SET tls2 { B|* }
NOTE BUSTER_TLS_T   tls2     -2.01883E-01 -1.38784E-01  3.45547E-01  1.26053E-02  3.36128E-02 -9.22257E-03
NOTE BUSTER_TLS_L   tls2      1.25664E+00  8.56587E-01  8.72291E-01  6.16143E-01 -7.38225E-01 -6.25366E-01
NOTE BUSTER_TLS_S   tls2     -1.32166E-02  1.45295E-01 -8.73965E-02  1.30264E-01  2.20430E-01  1.24629E-02 -6.42129E-02  2.72582E-02
NOTE BUSTER_TLS_O   tls2      2.19831E+01 -7.17259E+01  3.45078E+00
NOTE BUSTER_TLS_FIX tls2 ALL

 For final position, Geometry function summary
     term                           number     weight       rms        GooF      function     fn/numb
     ================================================================================================
     BOND bond lengths (angs)       5652       2.000      0.0075       0.372      1564.05       0.277
     ANGL bond angles (degs)        7670       2.000        1.00       0.599      5500.29       0.717
     TORS split-harmonic tors       1265       0.000       17.11       1.078         0.00       0.000
     SINTOR sinusoidal tors         1265       2.000       17.11                  2222.73       1.757
     PLAN general plane (angs)       963       5.000      0.0156       0.780      2931.16       3.044
     BCORrelations (angs^2)         5652      10.000       0.833       0.297      4994.80       0.884
     CONT (bad contacts)               0       5.000                                 0.00
     IDEAL(ideal-dist contact)      5049       4.000                             10317.53
     CHIRAL (gelly semiharmon)       740       5.000                                67.05
     SIM similarity restraint for NCS and/or TARGET                               9479.48
     OCCSUM restraint on sum of occupanies                                           0.00       as none defined
     DISTAN utility restraints interatomic distance                                  0.00       as none defined
     UTILANGLE utility angle restraints                                              0.00       as none defined
     UTILTOR utility restraints on torsion angles                                    0.00       as none defined
     Peptide omega torsion angles                           3.28 degs - monitor only
 
 WARNING: Have |delta/sigma| deviations > 5.0 sigma. Number of outliers for each term: 
 WARNING:         4 bond angles.  Worst is     5.5 sigs   135.51 degs B|28:C.B(ASN)=B|29:N(ASN)=B|29:CA(ASN)

 Weighted rms      0.0075         1.005  (temporary rms bond and angle for BUSTER to pick up!)
 Weighted intra-residual rms(bond) for StdProtein (excluding H)      0.0072
 Weighted intra-residual rms(bond) for Buffer     (excluding H)      0.0031
 Outputing geometry progress graph in plotmtv format to file:Geom_Res.mtv

 ############### Running final geometry/screen analysis (screen_final.txt)

 s/r gelly normal termination
```

Statistics and scale factors vs. cycle number
```

|  |  |  |
| --- | --- | --- |
|  | - Rxpct factors- <Log-Lik Gain>- GEOM Residuals- Ln Sigma_A_ | - Scale Factors- B Factors- Imperfection Factors |

```
   Final Geometry Information


---


### 2. Final results

Statistics and scale factors vs. cycle number
```

|  |  |  |
| --- | --- | --- |
|  | - Rxpct factors- <Log-Lik Gain>- GEOM Residuals- Ln Sigma_A_ | - Scale Factors- B Factors- Imperfection Factors |

```
   Average Figure of Merit
   Structure Factor Amplitudes


 # normal termination

  Program stopped.
```

closesok message: time spent in tree = 1744.70 (user) 42.10 (sys)

---

# Normal termination

```
 Resources usage summary:
     Time spent in client ...................  1744.71 (user)       42.12 (system)
     Time spent in scripts (    27 calls) ...     3.60 (user)        0.20 (system)
     Time spent in server ...................     0.00 (user)        0.00 (system)

     Max resident memory used (kb) .......... 521316
     Number of page faults .................. 0 (major)    1040622 (minor)
     Number of context switches ............. 1095540 (niced)    9223 (other)
```
